# Tailored growth of single-crystalline InP tetrapods

**DOI:** 10.1038/s41467-021-24765-7

**Published:** 2021-07-22

**Authors:** Youngsik Kim, Hyekyoung Choi, Yeunhee Lee, Weon-kyu Koh, Eunhye Cho, Taewan Kim, Hamin Kim, Yong-Hyun Kim, Hu Young Jeong, Sohee Jeong

**Affiliations:** 1 Department of Energy Science and Center for Artificial Atoms, Sungkyunkwan University (SKKU), Suwon, Republic of Korea; 2 Graduate School of Nanoscience and Technology, Korea Advanced Institute of Science and Technology (KAIST), Daejeon, Republic of Korea; 3 Department of Physics, Korea Advanced Institute of Science and Technology (KAIST), Daejeon, Republic of Korea; 4 UNIST Central Research Facilities, Ulsan National Institute of Science and Technology (UNIST), Ulsan, Republic of Korea

**Keywords:** Quantum dots, Synthesis and processing

## Abstract

Despite the technological importance of colloidal covalent III-V nanocrystals with unique optoelectronic properties, their synthetic process still has challenges originating from the complex energy landscape of the reaction. Here, we present InP tetrapod nanocrystals as a crystalline late intermediate in the synthetic pathway that warrants controlled growth. We isolate tetrapod intermediate species with well-defined surfaces of (110) and ($\bar{1}\bar{1}\bar{1}$) via the suppression of further growth. An additional precursor supply at low temperature induces $[\bar{1}\bar{1}\bar{1}]$-specific growth, whereas the [110]-directional growth occurs over the activation barrier of 65.7 kJ/mol at a higher temperature, thus finalizes into the (111)-faceted tetrahedron nanocrystals. We address the use of late intermediates with well-defined facets at the sub-10 nm scale for the tailored growth of covalent III-V nanocrystals and highlight the potential for the directed approach of nanocrystal synthesis.

## Abstract (web summary)

It is challenging to control the growth of colloidal III-V quantum dots, due to complex reaction pathways. Here, the authors isolate a single-crystalline tetrapod species as a late-stage intermediate and use it as a tailored-growth platform in colloidal synthesis.

## Introduction

Chemistry lies at the core of modern industry and technology owing to its precise atomic and molecular control of reaction pathways^1,2^. This has been particularly successful in the precise control of molecular structures for their specific purpose. The comprehensive resolution of such reaction pathways in terms of the reactants, intermediates, transition states, and products enables the development of new methods to create appropriate organic structures and materials^2^. On the other hand, colloidal semiconductor nanocrystals (NCs) have been exploited in academic and industrial sectors for more than 30 years, mainly via trial-and-error basis synthetic development owing to their unresolved reaction kinetics and thermodynamics^3–11^. Due to the co-existence of metastable states with similar energy, unraveling the reaction pathways in full detail has been difficult^12–16^.

Recently, a group of researchers revealed atomic information of II–VI, IV–VI, and III–V NCs through analytic methodologies and computational simulations^17–25^. To ultimately create a targeted structure at the sub-10-nm scale, one must identify, understand, and precisely control its reaction pathways with complete information on all possible intermediates. This is why research on molecule-like reaction intermediates of covalent III–V NCs has recently emerged and attracted considerable attention in the scientific community^26–28^.

**Fig. 1:**
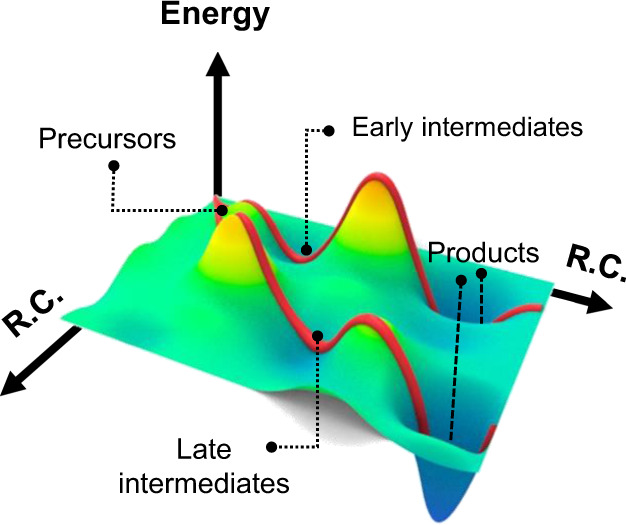
Schematic illustration of a complex energy landscape with multiple intermediates and transition states for growth reactions of inorganic NCs along with the reaction coordinates (R.C.). With various reaction coordinates, the early intermediates and late intermediates could exist depending on the reaction condition.

The reaction pathway of colloidal NCs is complicated as shown in Fig. 1, because various factors, such as the number of atoms, the composition, and the types and coverage of surface ligands change their energy landscape with a variety of intermediate species^14^. Here, we classify the intermediates as follows; the early intermediate is more similar to the reactants, and thus possesses a molecule-like amorphous structure; the late intermediate resembles products with a crystalline feature and well-defined facets, as marked in Fig. 1.

Recently, some early intermediates of III–V NCs such as magic-sized clusters have been isolated with full atomic information^22,28,29^. By bypassing the complicated monomer conversion steps, the use of early intermediates as reactants can be attempted to enhance the homogeneity of the products in III–V NC synthesis^28,30,31^. In contrast, product-like late intermediates, if identified, could serve as growth platform for targeted III–V NC structures.

Here, we firstly unravel the growth pathways of (111)-rich InP nanocrystals by identifying a key reaction intermediate, tetrapod, in the reaction. We then develop a synthetic route for mono-dispersed tetrapod intermediates with over 90% shape yield by catalyzing the nucleation, and thus suppressing further growth. The InP tetrapod reaction intermediates, with a single-crystalline zinc-blende structure have the well-defined atomic-scale surface information. Therefore, they can serve as the late intermediates for the directed growth to (111)-faceted tetrahedron-shaped NCs or other tetrapod NCs with thick or long arms by controlling the specific growth fronts of [110] and [$\bar{1}\bar{1}\bar{1}$] directions. Our late-intermediate strategy for the surface energy driven tailored growth of III–V NCs will certainly benefit to other NC synthesis that has unexplored reaction pathways.

## Results and discussion

### Observation of the tetrapod shape in early reaction stage of InP nanocrystal synthesis

**Fig. 2:**
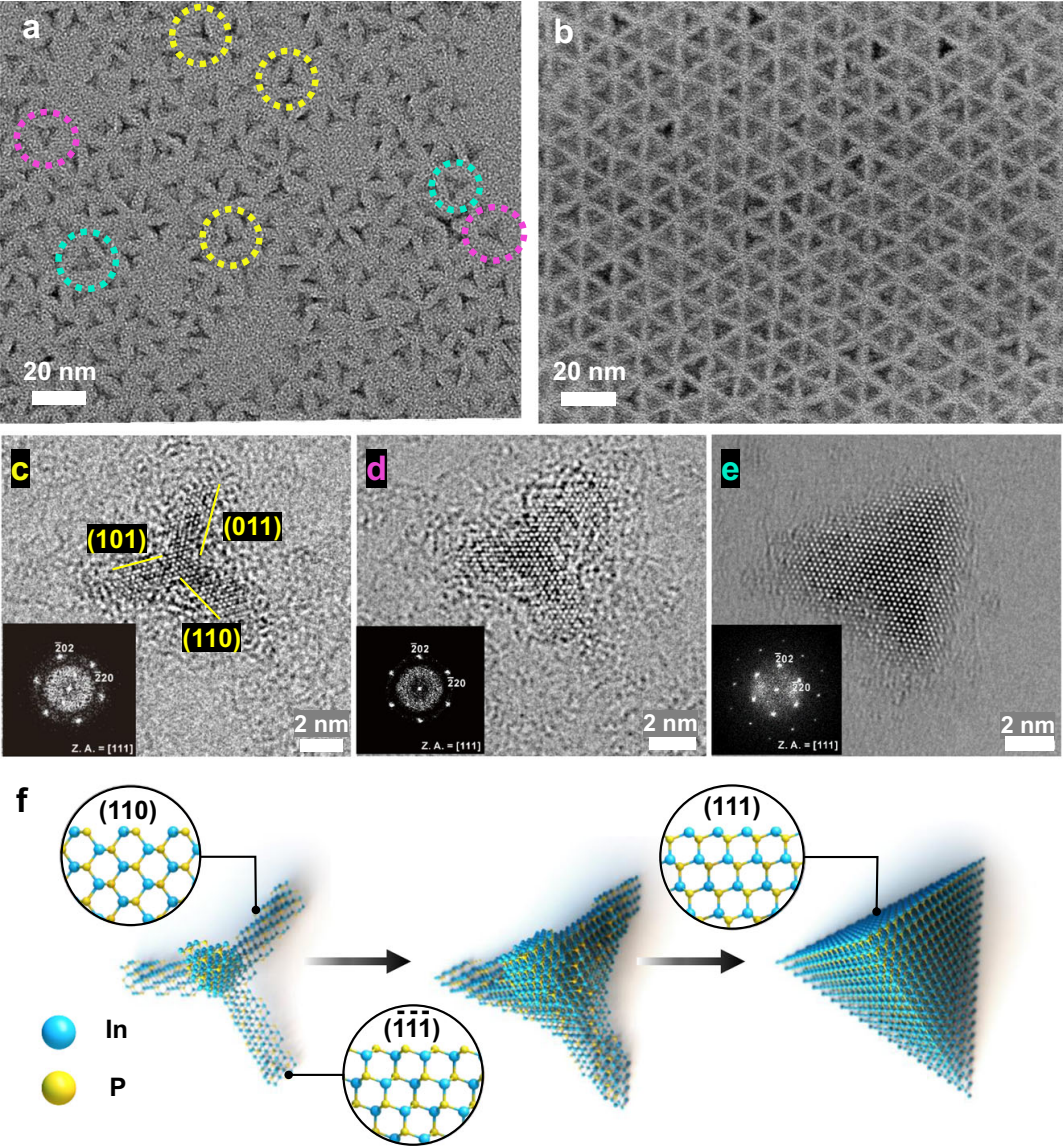
Structural snapshots of the anisotropic InP NCs. TEM images of **a** various InP tetrahedral nanostructures and **b** InP tetrahedron-shaped NCs. Several characteristic structures in **a** are marked with dotted-colored circles. **c–e** HR-TEM images (zone axis: [111]) of representative InP nanostructures found in **a** along the reaction pathway from the tetrapod to the tetrahedron, as schematically illustrated in **f** with the zinc-blende crystalline structure. One nanorod arm of the tetrapod grows along the [$\bar{1}\bar{1}\bar{1}$] direction covered with (110) facets. The tetrahedron is covered with four (111) facets. The insets in **c–e** show the fast Fourier transform (FFT) of the corresponding images.

Tetrahedron InP NCs are considered a suitable model for the surface study of colloidal III–V nanocrystals owing to their well-defined surfaces of only (111) facets passivated by chloride and oleylamine^19^. We discovered variously shaped nanostructures that co-exist in the synthesis of InP tetrahedron NCs, especially at the early stage of the reaction, as shown in Fig. 2a. Various thin and thick tetrapod-like InP NCs were observed in a transmission electron microscopy (TEM) image of the aliquot taken within 5 s after injection of the aminophosphine precursor at 300 °C. After 1 h under the same conditions, all of the particles were the tetrahedron shape, as shown in Fig. 2b. Tetrapod-like InP NCs were consistently observed at an early reaction stage at various reaction temperatures ranging from 170 to 300 °C (Supplementary Figs. 1–2). The early stage tetrapod grew into the tetrahedron as the reaction proceeded, except at 170 °C, where the tetrapod shape was retained until the end. Therefore, if they can be isolated from the reaction and stable in room temperature, the tetrapod-shaped InP NCs can be considered a reaction intermediate to the tetrahedron-shaped NCs.

The tetrahedral nanostructures can be classified into three types: thin tetrapods, thick tetrapods, and tetrahedrons (marked with yellow, pink, and sky-blue circles in Fig. 2a, respectively). More evidently, annular dark-field scanning TEM (ADF-STEM) images confirmed the shapes of the tetrapods and tetrahedrons based on the brighter contrast in the middle part of the InP NCs (Supplementary Fig. 3). High-resolution (HR) TEM images were obtained for the three types of intermediates, as shown in Fig. 2c–e. All of the HR-TEM images with fast Fourier transform (FFT) patterns clearly show {110} and {111}/{110} zinc-blende crystal lattice planes at the [111] zone axis (inset of Fig. 2c–e) and the [112] zone axis, respectively (Supplementary Figs. 4–5). Therefore, we propose that the thin tetrapod has arms exposed with {110} facets in the width direction and terminate with the {111} facet in the length direction and that the tetrahedron is enclosed by four {111} facets (Insets of Fig. 2f). In the In-rich crystal growth condition, the tetrahedron surface is known to be (111) terminated, which is exposed only by In atoms with one dangling bond^19^. As the (111) and ($\bar{1}\bar{1}\bar{1}$) surfaces are opposite in the zinc-blende crystal structure, the tetrapod end would be ($\bar{1}\bar{1}\bar{1}$), which is terminated with P atoms with one dangling bond or In atoms with three dangling bonds. Based on these findings, we speculate that the tetrapod grows into a tetrahedron such that the (111) facets emerge by filling the open space between the tetrapod arms, as schematically shown in Fig. 2f.

### Synthesis of InP tetrapods

Based on the collected atomic information for the tetrapod nanostructures, we hypothesized that (1) metastable tetrapod intermediates could be isolated by the growth suppression of a tetrahedron formation, and (2) the isolated tetrapod intermediates could be a novel platform for atomically controlled growth of III–V NCs based on surface energies of the (111) and (110) facets.

To suppress the continued growth of InP tetrahedrons, we additionally injected lithium bis(trimethylsilyl)amide (LiHMDS) to accelerate their precursor-conversion. LiHMDS is a well-known strong base (pKa of 30), allowing metal precursors to react with long-chain primary amines, which rapidly deprotonate RNH_2_ into RNHLi^32^. Similarly, we anticipate that InCl_3_ will turn to more reactive metal-amide complexes, as evidenced by the observation of In metal (XRD data for the reaction residue shown in Supplementary Fig. 6)^32–35^. Supplementary Fig. 7 depicts the UV-Vis spectra of the InP NCs synthesized at 250 °C under different LiHMDS concentrations. At a wavelength of *λ* = 413 nm, the intrinsic absorption coefficient of the InP NCs is independent of their size, and absorption from InP clusters smaller than [InP]_20_ is excluded^36–38^. As the amount of LiHMDS increases, the absorbance at *λ* = 413 nm increases and excitonic peaks show a blue shift. This indicates that LiHMDS increases the number of NCs via burst nucleation with a fast nucleation rate, which eventually leads to growth suppression through the rapid depletion of monomers. The suppression of the tetrapod-to-tetrahedron growth with the addition of LiHMDS is also confirmed by the decreased shape yield of tetrahedron from 81 to 20% (Supplementary Fig. 8b, c).

**Fig. 3:**
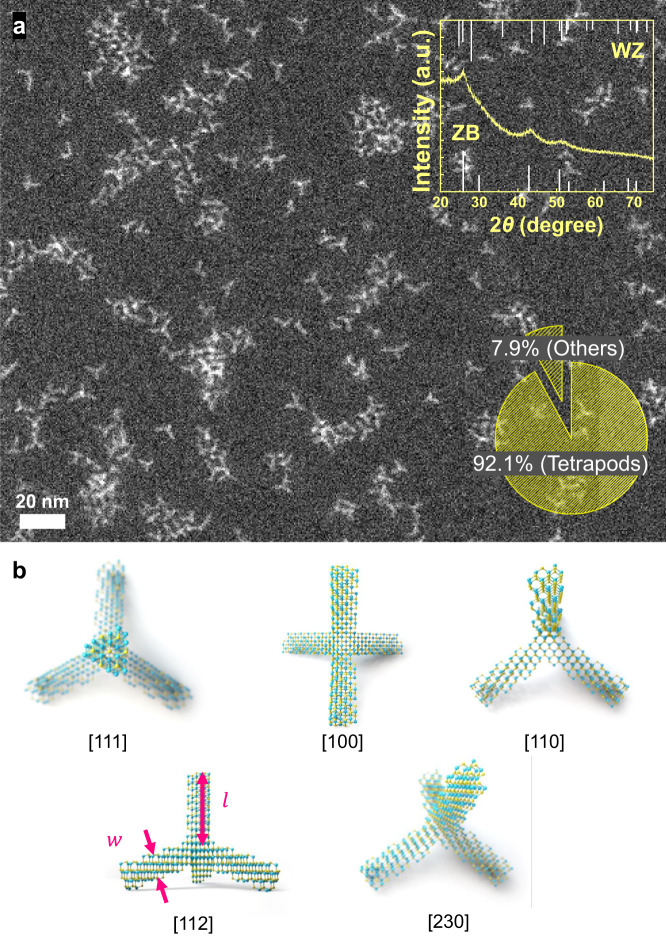
Isolated InP tetrapod intermediates. **a** STEM image of InP tetrapod NCs synthesized at 170 °C with the precursor-conversion accelerator. The XRD pattern in the inset confirms the zinc-blende structure (ZB zinc blende, WZ wurtzite). The pie chart in the inset represents the shape yield of the tetrapods. **b** Various shapes of tetrapod view at various zone axes. $l$ and $w$ represent the length and width of tetrapod arms, respectively.

As a result, we successfully synthesized tetrapod NCs with a near-unity shape yield (92.1%) that have a narrow size distribution of 2.2 ± 0.3 nm for the arm width and 6.8 ± 1.1 nm for the arm length (Fig. 3a and Supplementary Fig. 9). As the tetrapod shape could appear differently depending on the viewing angle, we take into account the view-angle dependent shapes (Fig. 3b) in the measurement of the size and the shape yield (see details in Supplementary Note 1). Notably, the InP tetrapod NCs in this study have a single-crystalline zinc-blende phase similar to the InP tetrahedron NCs as shown in the inset of Fig. 3a. Previously-reported tetrapod NCs typically have more than one crystal phase. For example, typical II–VI tetrapod NCs consist of a tetrahedral zinc-blend core and four wurtzite arms^39–41^. Furthermore, our InP tetrapods did not require further size-selective precipitation to enhance the uniformity, unlike the previous cases of III–V NCs with mixed shapes^42,43^. To generalize our idea for reaction intermediate isolation by catalyzing the monomer conversion that leads to the rapid depletion of the monomers, we used another precursor-conversion accelerator, that is, diisobutylaluminum hydride^44^ in our reaction. The shape conversion from tetrapod to tetrahedron again decreased to less than 14% (Supplementary Fig. 8d). Moreover, InAs tetrapod NCs were synthesized using the same reaction process with the precursor-conversion accelerator of LiHMDS (Supplementary Fig. 10).

**Fig. 4:**
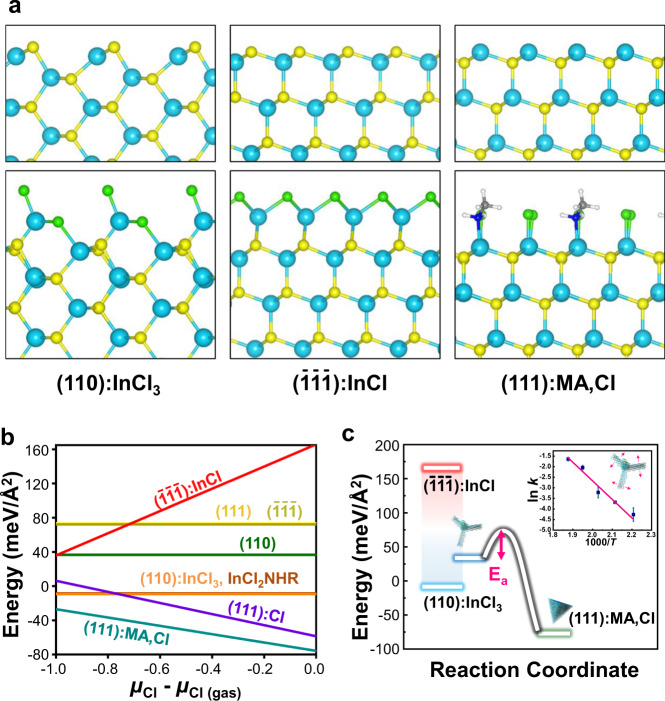
Density functional theory calculation of InP tetrapod surface energy depending on ligands on facets. **a** Side view of the bare (110), (111), and ($\bar{1}\bar{1}\bar{1}$) surface slab models (top) and ligand passivated surface slab models (bottom). The cyan, yellow, light green, blue, gray, and white balls represent In, P, Cl, N, C, and H atoms, respectively. **b** Surface formation energy of various surface models with passivating ligands depending on the Cl chemical potential. **c** Schematic of the reaction pathway from the late intermediate tetrapods to tetrahedron NCs. The inset in **c** shows the Arrhenius linear plot for the activation energy of tetrapod thickening toward the tetrahedron conversion. Error bars represent the standard deviation of ln *k* value extracted from absorbance spectra.

To understand the formation mechanism of the sub-10-nm sized single-crystalline tetrapods, we calculated the surface formation energies of the (111), (110), and ($\bar{1}\bar{1}\bar{1}$) surfaces with and without surface passivation ligands as a function of the chlorine chemical potential using density functional theory (DFT), as shown in Fig. 4a, b and listed in Supplementary Tables 1–2. For bare surfaces without any passivation, the (110) surface has the lowest surface energy of 36.6 meV/Å^2^. This is because the stoichiometric surface cation and anion atoms form a self-passivated reconstructed (110) surface. We found that the (110) surface could be further stabilized by the adsorption of the precursor molecule, InCl_3_ or its derivative, InCl_2_NHR. In case of InCl_3_, two Cl atoms passivate two surface In atoms, and the remaining InCl complex passivates two surface P atoms while it binds to a single P atom. In the presence of Cl and methylamine (MA), the (111) surface is appreciably stabilized by Cl and Cl/MA passivation while the ($\bar{1}\bar{1}\bar{1}$) surface shows a relatively high surface energy despite InCl passivation. Under our reaction conditions, InCl could be generated from InCl_3_ reduction by reducing agents such as aminosphosphine or amines^32,45–47^ (more details can be found in Supplementary Figs. 11 and 12). Our DFT results indicate that the tetrapod consisting of {110} and ($\bar{1}\bar{1}\bar{1}$) is a metastable intermediate, and the tetrahedron, consisting of (111):Cl or (111):MA,Cl, is thermodynamically the most stable one, as schematically drawn in Fig. 4c.

### Estimation of the activation energy barrier of [110] growth

To estimate the kinetic energy barrier of the transformation reaction from the intermediate tetrapod to the lowest-energy tetrahedron, we assumed that the [110] growth of tetrapod finally terminated into tetrahedrons though more complex chemistry may exist on tetrapod to tetrahedron transition. We reacted the isolated InP tetrapods with additional precursors at various reaction temperatures from 180 to 260 °C to particularly monitor the [110] growth of tetrapods. Assuming that the excitonic peaks in the absorbance spectra are predominantly determined by the width, rather than the length of the arms, a rate constant of the width growth was extracted and then fitted to the Arrhenius equation to obtain the energy barrier (see Methods section, Supplementary Figs. 13–14, and Supplementary Note 2 for details). The energy barrier was estimated to be 65.7 ± 6.2 kJ/mol, as shown in the inset of Fig. 4c. We surmise that this energy barrier may be associated with the chlorine activation from tetra(oleylamino)-phosphonium chloride, which is a key byproduct during amino-phosphine based InP NC synthesis^48^.

### Tailored growth from InP tetrapod late-intermediate

As we clearly identified the kinetic energy barrier in the tetrapod-to-tetrahedron reaction pathway, we were able to atomically control the growth reaction using the tetrapod as a late intermediate, e.g., to a more anisotropic InP NC with facet-specific growth control. Consequently, we realized tetrapods with long arms by further growing the [$\bar{1}\bar{1}\bar{1}$] facets from 8.3 ± 1.5 to 15.4 ± 3.1 nm (Supplementary Fig. 15). To do this, we slowly added the P precursor into the tetrapod intermediates and In precursors dispersed in oleylamine solution at a low reaction temperature of 170 °C, not to exceed the activation barrier (see Methods section). When the arm length becomes longer, a smaller shape yield of tetrapods was often observed (Supplementary Fig. 15). At higher temperatures, we obtained tetrahedron-shaped InP NCs (Supplementary Fig. 16) by overcoming the energy barrier and forming the (111) facets. Furthermore, we were able to tune the width of the InP tetrapod NCs from 2.3 to 2.9 nm, selectively growing the (110) facets from the long-arm tetrapod intermediates, by adjusting the reaction temperature without introducing additional precursors (see Methods section and Supplementary Fig. 17).

Our elemental analyses based on X-ray photoelectron spectroscopy measurements indicate that the In/P ratio of the representative short- and long-arm tetrapods is 1.3 and 1.2, respectively, which is smaller than that of the InP tetrahedron NCs 1.5 (Supplementary Table 3). The adsorption of InCl_3_ and InCl_x_(NHR)_y﻿_ on stoichiometric (110) may be responsible for the higher In/P ratio and relatively high Cl and N contents in the tetrapods from XPS analysis in Supplementary Table 3.

### Width- and length-tailored InP tetrapods

**Fig. 5:**
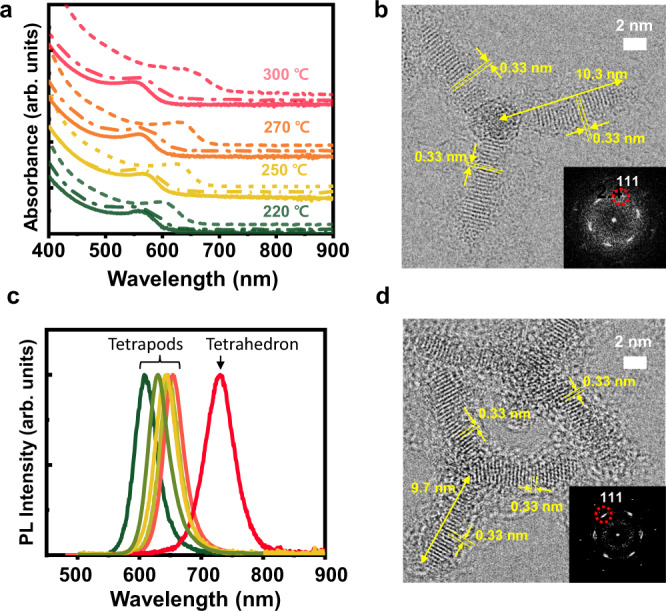
Width- and length-tailored tetrapods. **a** Absorption spectra of the atomically tailored InP tetrapod NCs: the solid lines represent as-isolated tetrapods; dashed-dotted lines represent length-controlled tetrapods that are additionally [$\bar{1}\bar{1}\bar{1}$] grown from as-isolated tetrapods; and short-dashed lines represent width-controlled tetrapods obtained after annealing at various temperatures. **b** HR-TEM image of a tetrapod NC with additional NC growth along both the [110] and [$\bar{1}\bar{1}\bar{1}$] directions from as-isolated tetrapod intermediates. **c** Photoluminescence spectra of the HF-treated InP tetrapods with various arm widths and HF-treated tetrahedron NCs. **d** HR-TEM image of the tetrapod NC after HF treatment for 190 min (corresponding photoluminescence spectrum shown as the yellow line in **c**).

Figure 5a shows the changes in first excitonic peaks of the various length- and width-controlled InP tetrapod NCs. We found that the optical bandgaps of the tetrapods were mainly determined by the width rather than the length of the arms (Fig. 5a and Supplementary Figs. 17–19). We note that the first excitonic peaks from the short- and long-arm tetrapods almost overlap whereas the exciton peak continually red-shifts up to 100 nm from thick-arm tetrapods when heated to 300 °C because of the selective (110) width growth.

Figure 5b shows the HR-TEM image of a tetrapod with a length of 10.3 nm with atomic resolution. The (111) lattice plane was clearly visible, likely due to the three arms attached to the TEM grid. Accordingly, the FFT pattern in the inset of Fig. 5b shows six {111} diffraction spots. The measured distance of the (111) plane was 3.3 Å, which is very close to that of the zinc-blende InP single-crystalline structure. We confirmed that, regardless of the arm lengths and widths of the InP tetrapod NCs, the X-ray diffraction (XRD) patterns matched those of the InP zinc-blende structure (Supplementary Fig. 20).

As we suppressed the inhomogeneity of InP crystals, our pure tetrapods may benefit optoelectronic characteristics, distinct from previously prevalent mixed-crystalline II–VI tetrapod NCs^39,49,50^. HF treatment with UV irradiation on as-synthesized tetrapods resulted in a dramatic increase in photoluminescence (PL) emission^51–54^ (Supplementary Fig. 21) while retaining their structure as a zinc blende seen in HR-TEM image (Fig. 5d and Supplementary Fig. 22) and XRD spectra (Supplementary Fig. 23). XPS analyses show that In 3d binding energy shifted to a higher energy indicating the formation of In–F or In–O. The increased F 1s peak in XPS indicates the F passivation of InP surfaces (Supplementary Fig. 24)^51^. Indeed, our DFT calculations show that the F passivation of the (110) surface clears the surface states, which can serve as trap states in the quantum-confinement regime (Supplementary Fig. 25). Thickness-controlled tetrapods with HF treatment (see Methods section)^51,52^ show PL emissions ranging from 608 to 654 nm with 36–42 nm PL FWHM, while the tetrahedrons show PL of up to 730 nm with 55 nm FWHM (Fig. 5c). We achieved as low as 36 nm (0.10 eV) of FWHM at the maximum PL of 646 nm (Supplementary Fig. 26). This is comparable to the narrowest PL FWHM of 35.9 nm at 630 nm in the InP NCs^11^.

A shape-dependent electronic structure at the confinement regime has long been of interest in obtaining superior or tailored optical properties for NCs, but is severely limited by the synthetic availability of various well-defined shapes at the nanoscale^55–58^. Our III–V single-crystalline tetrapods could be a testbed for studying complex exciton structures with complex geometries.

In conclusion, we isolated late intermediate single-crystalline tetrapods with well-defined faceting in the complex reaction pathways in III–V nanomaterial synthesis. The late intermediate tetrapods can be transformed into thermodynamically stable tetrahedron NCs by overcoming the reaction energy barrier. By controlling the reaction temperature and the amount of precursors, we were able to selectively grow ($\bar{1}\bar{1}\bar{1}$) and (110) facets. Based on the atomic information of the late intermediate, we could predict and realize the designed growth during wet synthesis of III–V NCs, which had been largely limited owing to the lack of a well-defined growth platform. We suggest that this finding will bring insights in the field of the intermediate-involved directed approach of nanocrystal synthesis.

## Methods

### Chemicals

Indium chloride (InCl_3_, 98%), tris(dimethylamino)phosphine ((Me_2_N)_3_P), 97%), oleylamine (70%), lithium bis(trimethylsilyl)amide (or lithium hexamethyldisilazide, LiHMDS, 97%), diisobutylaluminum hydride (DIBAL-H, 1.0 M in toluene), and hydrofluoric acid (HF, 48 wt% in H_2_O) were purchased from Sigma-Aldrich. Tris(dimethylamino)arsine ((Me_2_N)_3_As), 99%) was purchased from Strem. Unless otherwise stated, all chemicals were used without further purification. Degassed oleylamine (D-OLA) was prepared by degassing under vacuum (~1.0 Torr) at 90 °C for 60 min.

### Synthesis of InP tetrahedron nanocrystals

InCl_3_ (0.2257 g, 1 mmol) and oleylamine (5 mL) were degassed at 90 °C under vacuum (~1.0 Torr) for 1 h. The solution was heated to the injection temperature (170–300 °C) under an N_2_ gas atmosphere. Separately, a mixed solution of 0.187 mL (1 mmol) of (Me_2_N)_3_P and 0.5 mL of D-OLA was prepared for P injection in the glove box. The prepared solution was rapidly injected into the mixed solution of InCl_3_ and oleylamine at temperature ranging from 170 to 300 °C, depending on the target size of the NCs, and the reaction temperature was maintained for 60 min. As-grown InP NCs were isolated by precipitation once with acetone and once with methyl acetate and redispersed in hexane for further analysis.

### Synthesis of InP tetrapod NCs with LiHMDS

The degassing process of InCl_3_ and oleylamine was the same as the synthesis of the InP tetrahedron nanocrystals. The degassed solution was heated to an injection temperature of 170 °C under an N_2_ atmosphere. The injection solution was prepared by mixing 0.016 g (0.1 mmol) of LiHMDS in 0.5 mL of oleylamine and 0.187 mL (1 mmol) of (Me_2_N)_3_P in a glovebox. The prepared solution was injected into InCl_3_ in oleylamine at 170 °C (Fig. 3a) and maintained for 60 min. For the precipitation, the reaction mixture was transfered from flask to 50 ml of conical tube, followed by centrifuged 6000 rpm for 5 min. Supernatant and precipitates were separated, then isolated supernatant is washed same as for the InP tetrahedron NCs washing process. To confirm the role of LiHMDS, we varied the amount of LiHMDS from 0.0 to 0.4 mmol per 1.0 mmol of InCl_3_ at reaction temperature of 250 °C, and measured its absorption spectrum and TEM (Supplementary Figs. 7 and 8a–c).

### Synthesis of InP tetrapod NCs with DIBAL-H

InP tetrapod NCs with DIBAL-H were prepared following the same procedure for the InP tetrapod NCs with 0.4 mmol of DIBAL-H at 250 °C.

### Synthesis of InAs tetrapod NCs

All the experimental processes are the same as the InP tetrapod NCs with LiHMDS, except for the preparation of As injection solution and reaction temperature. The injection solution was prepared with 0.092 mL of (Me_2_N)_3_As (0.5 mmol) and 0.016 g (0.1 mmol) of LiHMDS in 0.5 mL of D-OLA. The solution was heated to 40 °C for 10 min to remove gases from transamination. Prepared As solution was rapidly injected into the InCl_3_ in OLA at 220 °C, and the temperature was maintained for 60 min.

### Determination of the activation energy barrier for the [110] growth

Preparation of InP tetrapod (solution A): InP tetrapods were synthesized at 170 °C using LiHMDS with 10-fold scale-up; besides this, the synthesis was the same as the InP tetrapod synthesis. This was followed by precipitation and re-dispersion in hexane to 0.07 g/mL. No LiHMDS remained in the solution.

Preparation of In precursor solution (solution B): InCl_3_ (0.1354 g, 0.6 mmol) in 20 mL of oleylamine was degassed at 90 °C under vacuum (~1.0 Torr) for 60 min.

Growth of [110] from isolated InP tetrapod: As-prepared InP tetrapod (0.14 g, solution A) was injected into the solution B at 90 °C, and heated to the target reaction temperature from 180 to 260 °C under N_2_. At the elevated temperature, 0.281 mL (1.5 mmol) of (Me_2_N)_3_P solution was injected. The aliquots were taken to extract the reaction rate depending on the reaction temperature.

### Atomically controlled [$\bar{{{{\bf{1}}}}}\bar{{{{\bf{1}}}}}\bar{{{{\bf{1}}}}}$]/[110] growth of InP tetrapods

All the experimental processes are similar to the “Determination of the activation energy barrier for [110] growth” except the concentration of In and P sources, and the injection method. In the “Determination of the activation energy barrier for [110] growth”, we used a highly diluted In and P precursors with the hot injection method to obtain the activation energy by preventing additional nucleation. Here we used a sufficient precursors for [$\bar{1}\bar{1}\bar{1}$]/[110] growth.

InP tetrapods were synthesized at 170 °C using LiHMDS with 10-fold scale-up; besides this, the synthesis was the same as the InP tetrapod synthesis. This was followed by precipitation and re-dispersion in hexane to 0.07 g/mL. No LiHMDS remained in the solution. Separately, 0.2257 g (1.0 mmol) of InCl_3_ in 5 mL of oleylamine was loaded into a three-neck flask, and the solution was degassed at 90 °C under vacuum (~1.0 Torr). Then, 0.14 g of InP tetrapods in hexane was injected into the InCl_3_ solution at 90 °C and heated to 170 °C under N_2_ atmosphere. (Me_2_N)_3_P (0.937 mL, 5 mmol) in 3.063 mL of oleylamine was slowly injected at the rate of 0.05 mL/min (for the tetrahedron InP, we used 250 °C, Supplementary Fig. 16). The reaction solution was additionally heated for 40 min at the same temperature after the complete introduction of the P precursor solution. TEM analysis showed that the obtained tetrapods had long arms via selective [$\bar{1}\bar{1}\bar{1}$] growth (Supplementary Figs. 15 and 19). For further growth along the [110] direction, the reaction solution was heated to various temperatures of 220, 250, 270, and 300 °C, and maintained for 60 min (Fig. 5a and Supplementary Figs. 17–18). The precipitation process was the same as that followed for the InP tetrahedral NCs.

### HF-treated InP tetrapod NCs

The [$\bar{1}\bar{1}\bar{1}$]/[110] grown InP tetrapod NCs (described above) were treated with the HF solution as previously described with slight modifications^51,53^. Diluted HF solution was prepared by mixing with 5 mL of butanol, 0.565 mL of 48 wt% HF solution, and 0.065 mL of deionized water. Separately, 0.07 g of [$\bar{1}\bar{1}\bar{1}$]/[110] grown InP tetrapod NCs was dispersed in a mixed solution of 7 mL of hexane, 5 mL of butanol, and 0.05 g of TOPO. The mixed solution was stirred vigorously. Subsequently, 0.3 mL of the diluted HF solution was added to the tetrapod solution and irradiated with UV light. After 190 min, the reaction was stopped, and the solution was precipitated using butanol.

### Optical spectra

Absorption spectra were measured using a UV-Vis spectrometer (Evolution 201, Thermo Scientific). Room-temperature photoluminescence quantum yields were measured using an absolute photoluminescence quantum yield spectrometer (C9920-02, Hamamatsu).

### Structural analysis

X-ray diffraction (XRD, Empyrean, PANalytical) was used to study the crystal structure of the InP NCs. The size and shape of the NCs were determined by spherical aberration-corrected TEM (JEOL, JEM-ARM 200 F) with an acceleration voltage of 200 kV at the National NanoFab Center, Republic of Korea. High-resolution TEM images (Figs. 2c–e and 5b, d and Supplementary Figs. 3–5) were taken by spherical aberration-corrected TEM (FEI Titan3 G2 60–300) with an acceleration voltage of 80 kV at Ulsan National Institute of Science and Technology (UNIST). To obtain high signal-to-noise ratio images, we used a single layer of graphene as a supporting film for TEM imaging. As the NCs could be damaged by the e-beam during TEM measurement at high magnification, we evaluated the arm length and width of the tetrapods with low-magnification images^59^.

### Elemental analysis

X-ray photoemission spectroscopy was performed using the Sigma Probe model (Thermo VG Scientific).

### Computational details

We performed first-principles density functional theory (DFT) calculations by employing all-electron-like projector-augmented wave potentials^60^ and Perdew–Burke–Ernzerhof (PBE) exchange-correlation functionals^61^ as implemented in the Vienna Ab Initio Simulation Package (VASP)^62,63^. A plane-wave energy cutoff of 550 eV was used, and all the atoms were fully relaxed until the atomic forces and electronic energies were <0.02 eV/Å and 10^−4^ eV, respectively.

For the non-polar (110) surface, with the same atomic structure as the front and back surfaces in the atomic slab model, as shown in Supplementary Fig. 27a, the absolute surface energy is simply the average of the total surface energy:

1 $$\gamma =\frac{1}{2}\frac{{E}_{{{{\rm{slab}}}}}-{E}_{{{{\rm{bulk}}}}}}{A}$$

where *E*_slab_ and *E*_bulk_ are the DFT total energies of the slab model and the corresponding bulk system, respectively, and *A* is the surface area of the front surface.

In contrast, when the compositions of the front and back surfaces are different, e.g., in the (111)/($\bar{1}\bar{1}\bar{1}$) surface slab model, the surface energy obtained from Eq. (1) is the average of the front (111) and back ($\bar{1}\bar{1}\bar{1}$) surfaces; therefore, the average is not the exact absolute value of each surface. Accordingly, to obtain the absolute surface energy of the polar facet, we used different-sized wedge models passivated by pseudohydrogen atoms based on the electron-counting rule (Supplementary Fig. 27b, c)^64^. First, to obtain the absolute energy of (100) surface, we designed the wedge models exposing (100) and (110) surfaces (Supplementary Fig. 27b) with 9–10 atoms on the (100) surface. Then, we constructed the wedge models corresponding to Fig. 27c with 6–10 atoms in the (111) surface. Using the non-polar (110) surface energy from the slab calculation (Supplementary Fig. 27a), we were able to calculate the absolute surface energies of (100) and (111) from the wedge model (Supplementary Fig. 27b, c). The calculated absolute surface energies of the (111) and ($\bar{111}$) surfaces converged to within approximately 0.4 meV/Å^2^. We employed (1 × 1) surface slab models with the (12 × 12 × 1) k-points sampling, except for the chlorine-amine co-passivated (2 × 2) surface. The wedge model with (1 × 1 × 12) k-points sampling had a triangle-shaped cross-section and infinitely periodicity along the z-direction.

To determine the surface stability, we calculated the surface formation energy with ligand coordination as a function of the chlorine chemical potential. The formation energy was defined as follow:

2 $${E}_{{{{\rm{Formation}}}}}=E({{{\rm{surface}}}}/{{{\rm{ligand}}}})-E\left({{{\rm{surface}}}}\right)-E({{{\rm{ligand}}}})$$

where *E* is the DFT total energy of each system. The chlorine chemical potential was referenced to a free chlorine gas molecule.

## Supplementary information

Supplementary Information

## Data Availability

The data related with this paper is available through the corresponding author upon reasonable request.

## References

[1] McBain JW (1923). Chemistry and modern life. Science.

[2] McMurry, J. *Cengage Learning* (Cengage Learning, 2010).

[3] Brus LE (1984). Electron-electron and electron-hole interactions in small semiconductor crystallites: the size dependence of the lowest excited electronic state. J. Chem. Phys..

[4] Efros AL, Efros AL (1982). Interband light absorption in semiconductor spheres. Sov. Phys. Semicond. USSR.

[5] Murray CB, Norris DJ, Bawendi MG (1993). Synthesis and characterization of nearly monodisperse CdE (E = S, Se, Te) semiconductor nanocrystallites. J. Am. Chem. Soc..

[6] Bruchez M, Moronne M, Gin P, Weiss S, Alivisatos AP (1998). Semiconductor nanocrystals as fluorescent biological labels. Science.

[7] Klimov VI (2007). Single-exciton optical gain in semiconductor nanocrystals. Nature.

[8] Achermann M (2004). Energy-transfer pumping of semiconductor nanocrystals using an epitaxial quantum well. Nature.

[9] Tang J (2011). Colloidal-quantum-dot photovoltaics using atomic-ligand passivation. Nat. Mater..

[10] Kovalenko MV (2015). Prospects of nanoscience with nanocrystals. ACS Nano.

[11] Won Y-H (2019). Highly efficient and stable InP/ZnSe/ZnS quantum dot light-emitting diodes. Nature.

[12] Billinge SJL, Levin I (2007). The problem with determining atomic structure at the nanoscale. Science.

[13] Wang F, Richards VN, Shields SP, Buhro WE (2014). Kinetics and mechanisms of aggregative nanocrystal growth. Chem. Mater..

[14] Lee J, Yang J, Kwon SG, Hyeon T (2016). Nonclassical nucleation and growth of inorganic nanoparticles. Nat. Rev. Mater..

[15] McVey BFP (2019). Unraveling the role of zinc complexes on indium phosphide nanocrystal chemistry. J. Chem. Phys..

[16] Vikram A (2020). Mechanistic insights into size-focused growth of indium phosphide nanocrystals in the presence of trace water. Chem. Mater..

[17] Choi H, Ko JH, Kim YH, Jeong S (2013). Steric-hindrance-driven shape transition in PbS quantum dots: understanding size-dependent stability. J. Am. Chem. Soc..

[18] Woo JY (2014). Ultrastable PbSe nanocrystal quantum dots via in situ formation of atomically thin halide adlayers on PbSe(100). J. Am. Chem. Soc..

[19] Kim K (2016). Halide-amine co-passivated indium phosphide colloidal quantum dots in tetrahedral shape. Angew. Chem. Int. Ed..

[20] Voznyy O, Sargent EH (2014). Atomistic model of fluorescence intermittency of colloidal quantum dots. Phys. Rev. Lett..

[21] Kim D, Kim DH, Lee JH, Grossman JC (2013). Impact of stoichiometry on the electronic structure of PbS quantum dots. Phys. Rev. Lett..

[22] Gary DC (2016). Single-crystal and electronic structure of a 1.3 nm indium phosphide nanocluster. J. Am. Chem. Soc..

[23] Williamson CB (2019). Chemically reversible isomerization of inorganic clusters. Science.

[24] Kim JY, Steeves AH, Kulik HJ (2017). Harnessing organic ligand libraries for first-principles inorganic discovery: indium phosphide quantum dot precursor design strategies. Chem. Mater..

[25] Tamang S, Lincheneau C, Hermans Y, Jeong S, Reiss P (2016). Chemistry of InP nanocrystal syntheses. Chem. Mater..

[26] Reiss P (2016). Synthesis of semiconductor nanocrystals, focusing on nontoxic and earth-abundant materials. Chem. Rev..

[27] Kim Y (2020). III–V colloidal nanocrystals: control of covalent surfaces. Chem. Sci..

[28] Gary DC, Terban MW, Billinge SJL, Cossairt BM (2015). Two-step nucleation and growth of Inp quantum dots via magic-sized cluster intermediates. Chem. Mater..

[29] Kwon Y (2020). Evolution from unimolecular to colloidal-quantum-dot-like character in chlorine or zinc incorporated InP magic size clusters. Nat. Commun..

[30] Tamang S, Lee S, Choi H, Jeong S (2016). Tuning size and size distribution of colloidal InAs nanocrystals via continuous supply of prenucleation clusters on nanocrystal seeds. Chem. Mater..

[31] Zhao Q, Kulik HJ (2018). Electronic structure origins of surface-dependent growth in III-V quantum dots. Chem. Mater..

[32] Miller RC, Neilson JR, Prieto AL (2020). Amide-assisted synthesis of iron germanium sulfide (Fe2GeS4) nanostars: the effect of LiN(SiMe3)2 on precursor reactivity for favoring nanoparticle nucleation or growth. J. Am. Chem. Soc..

[33] Tamang S, Kim K, Choi H, Kim Y, Jeong S (2015). Synthesis of colloidal InSb nanocrystals via in situ activation of InCl3. Dalton Trans..

[34] Grigel V, Dupont D, De Nolf K, Hens Z, Tessier MD (2016). InAs colloidal quantum dots synthesis via aminopnictogen precursor chemistry. J. Am. Chem. Soc..

[35] He M, Protesescu L, Caputo R, Krumeich F, Kovalenko MV (2015). A general synthesis strategy for monodisperse metallic and metalloid nanoparticles (In, Ga, Bi, Sb, Zn, Cu, Sn, and Their Alloys) via in situ formed metal long-chain amides. Chem. Mater..

[36] McMurtry BM (2020). Continuous nucleation and size dependent growth kinetics of indium phosphide nanocrystals. Chem. Mater..

[37] Friedfeld MR, Johnson DA, Cossairt BM (2019). Conversion of InP clusters to quantum dots. Inorg. Chem..

[38] Tessier MD, Dupont D, De Nolf K, De Roo J, Hens Z (2015). Economic and size-tunable synthesis of InP/ZnE (E = S, Se) colloidal quantum dots. Chem. Mater..

[39] Manna L, Milliron DJ, Meisel A, Scher EC, Alivisatos AP (2003). Controlled growth of tetrapod-branched inorganic nanocrystals. Nat. Mater..

[40] Lim J (2013). Controlled synthesis of CdSe tetrapods with high morphological uniformity by the persistent kinetic growth and the halide-mediated phase transformation. Chem. Mater..

[41] Fiore A (2009). Tetrapod-shaped colloidal nanocrystals of II−VI semiconductors prepared by seeded growth. J. Am. Chem. Soc..

[42] Kwon Y, Bang G, Kim J, Agnes A, Kim S (2020). Synthesis of InP branched nanostructures by controlling the intermediate nanoclusters. J. Mater. Chem. C.

[43] Ahrenkiel SP (2003). Synthesis and characterization of colloidal InP quantum rods. Nano Lett..

[44] Srivastava V, Dunietz E, Kamysbayev V, Anderson JS, Talapin DV (2018). Monodisperse InAs quantum dots from aminoarsine precursors: understanding the role of reducing agent. Chem. Mater..

[45] Tessier MD (2016). Aminophosphines: a double role in the synthesis of colloidal indium phosphide quantum dots. J. Am. Chem. Soc..

[46] Mourdikoudis S, Liz-Marzán LM (2013). Oleylamine in nanoparticle synthesis. Chem. Mater..

[47] Fowles, G. W. A. *Reaction of Metal Halides with Ammonia and Aliphatic Amines*. 1–36 (John Wiley & Sons, Inc., 1964).

[48] Buffard A (2016). Mechanistic insight and optimization of inp nanocrystals synthesized with aminophosphines. Chem. Mater..

[49] Wang J (2013). Colloidal synthesis of Cu2SnSe3 tetrapod nanocrystals. J. Am. Chem. Soc..

[50] Enright MJ (2020). Seeded growth of nanoscale semiconductor tetrapods: generality and the role of cation exchange. Chem. Mater..

[51] Adam S (2005). The effect of nanocrystal surface structure on the luminescence properties: photoemission study of HF-etched InP nanocrystals. J. Chem. Phys..

[52] Talapin DV (2002). Etching of colloidal InP nanocrystals with fluorides: photochemical nature of the process resulting in high photoluminescence efficiency. J. Phys. Chem. B.

[53] Mićić OI, Sprague J, Lu Z, Nozik AJ (1996). Highly efficient band‐edge emission from InP quantum dots. Appl. Phys. Lett..

[54] Siramdas R, McLaurin EJ (2017). InP nanocrystals with color-tunable luminescence by microwave-assisted ionic-liquid etching. Chem. Mater..

[55] Shabaev A, Efros AL (2004). 1D exciton spectroscopy of semiconductor nanorods. Nano Lett..

[56] Puangmali T, Califano M, Harrison P (2010). Monotonic evolution of the optical properties in the transition from three- to quasi-two-dimensional quantum confinement in InAs nanorods. J. Phys. Chem. C.

[57] Sercel PC, Efros AL (2018). Band-edge exciton in CdSe and other II-VI and III-V compound semiconductor nanocrystals - revisited. Nano Lett..

[58] Nagamine G (2018). Evidence of band-edge hole levels inversion in spherical CuInS2 quantum dots. Nano Lett..

[59] Williams, D. B. & Carter, C. B. *Transmission Electron Microscopy: A Textbook for Materials Science* (Springer Science and Business Media, 2009).

[60] Blöchl PE (1994). Projector augmented-wave method. Phys. Rev. B.

[61] Perdew JP, Burke K, Ernzerhof M (1996). Generalized gradient approximation made simple. Phys. Rev. Lett..

[62] Kresse G, Furthmüller J (1996). Efficient iterative schemes for Ab initio total-energy calculations using a plane-wave basis set. Phys. Rev. B.

[63] Kresse G, Furthmüller J (1996). Efficiency of ab-initio total energy calculations for metals and semiconductors using a plane-wave basis set. Comput. Mater. Sci..

[64] Zhang SB, Wei SH (2004). Surface energy and the common dangling bond rule for semiconductors. Phys. Rev. Lett..

