# Supplementary material for: Tailored growth of single-crystalline InP tetrapods
Source: Nat Commun. 2021 Jul 22;12:4454. doi: 10.1038/s41467-021-24765-7 (PMC8298524; doi:10.1038/s41467-021-24765-7)
Supplement: Supplementary file 1 — Supplementary Information [file 41467_2021_24765_MOESM1_ESM.pdf]

Supplementary Information

Tailored growth of single-crystalline InP tetrapods

Authors: Youngsik Kim[1]‡, Hyekyoung Choi[1]‡, Yeunhee Lee[2]‡, Weon-kyu Koh[1], Eunhye Cho[1], Taewan Kim[1], Hamin Kim[1], Yong-Hyun Kim[2,3]*, Hu Young Jeong[4]*, and Sohee Jeong[1]*

Affiliations:

[1]Department of Energy Science and Center for Artificial Atoms, Sungkyunkwan University (SKKU), Suwon 16419, Republic of Korea

[2]Graduate School of Nanoscience and Technology, Korea Advanced Institute of Science and Technology (KAIST), Daejeon 34141, Republic of Korea

[3]Department of Physics, Korea Advanced Institute of Science and Technology (KAIST), Daejeon 34141, Republic of Korea

[4]UNIST Central Research Facilities, Ulsan National Institute of Science and Technology (UNIST), Ulsan 44919, Republic of Korea

[†]These authors contributed equally to this work. Youngsik Kim, Hyekyoung Choi, and Yeunhee Lee

*These authors jointly supervised this work: Sohee Jeong, Hu Young Jeong, and Yong-Hyun Kim *e-mail: s.jeong@skku.edu (S.J.); hulex@unist.ac.kr (H.Y.J.); yong.hyun.kim@kaist.ac.kr (Y.-H.K.)

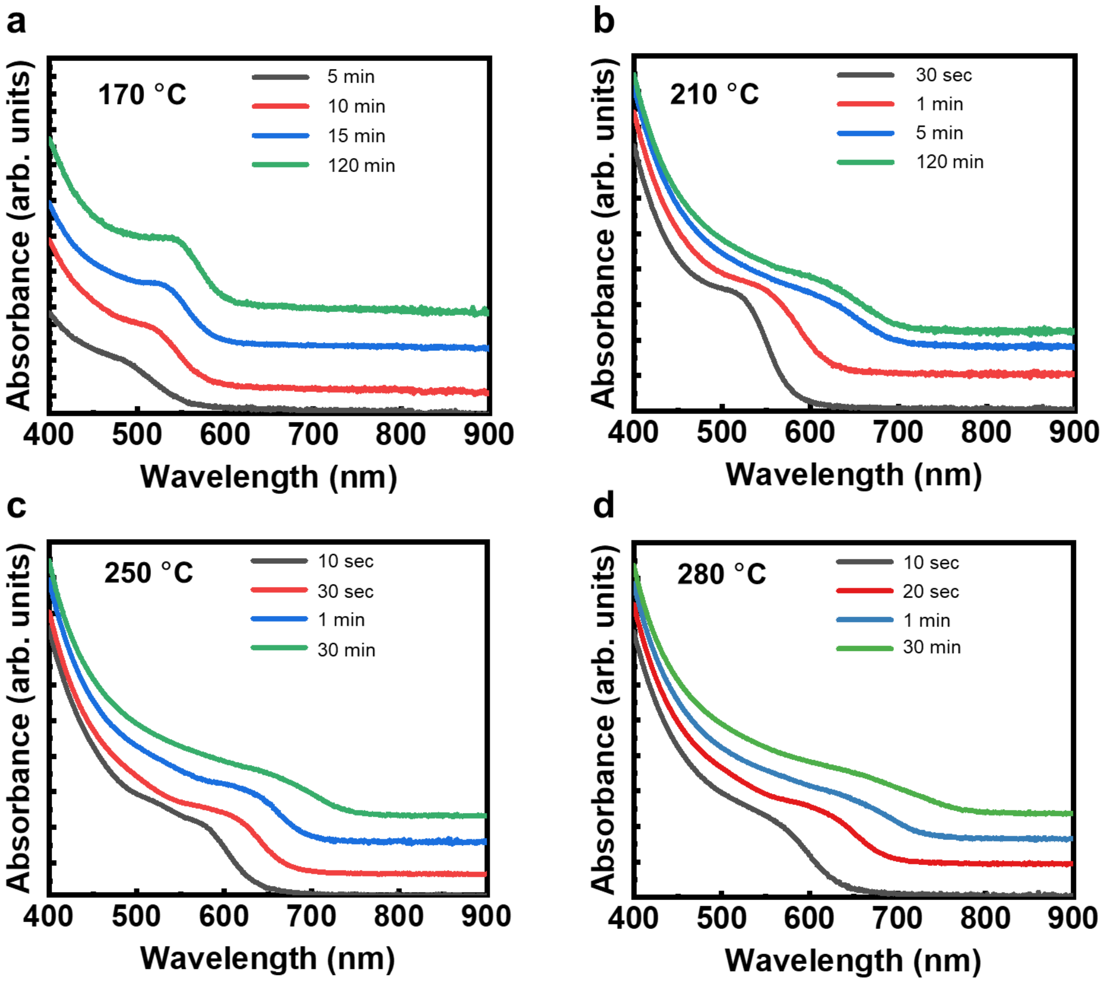

Supplementary Figure 1. Absorbance spectra of the aliquots taken during the reaction at **a** 170, **b** 210, **c** 250, and **d** 280 °C to investigate the existence of the reaction intermediates at the early stage of InP formation using $InCl_3$, oleylamine, and tris(dimethylamino)phosphine as reaction precursors. Considering the different reaction rates depending on the reaction temperature, aliquots were taken at various reaction times.

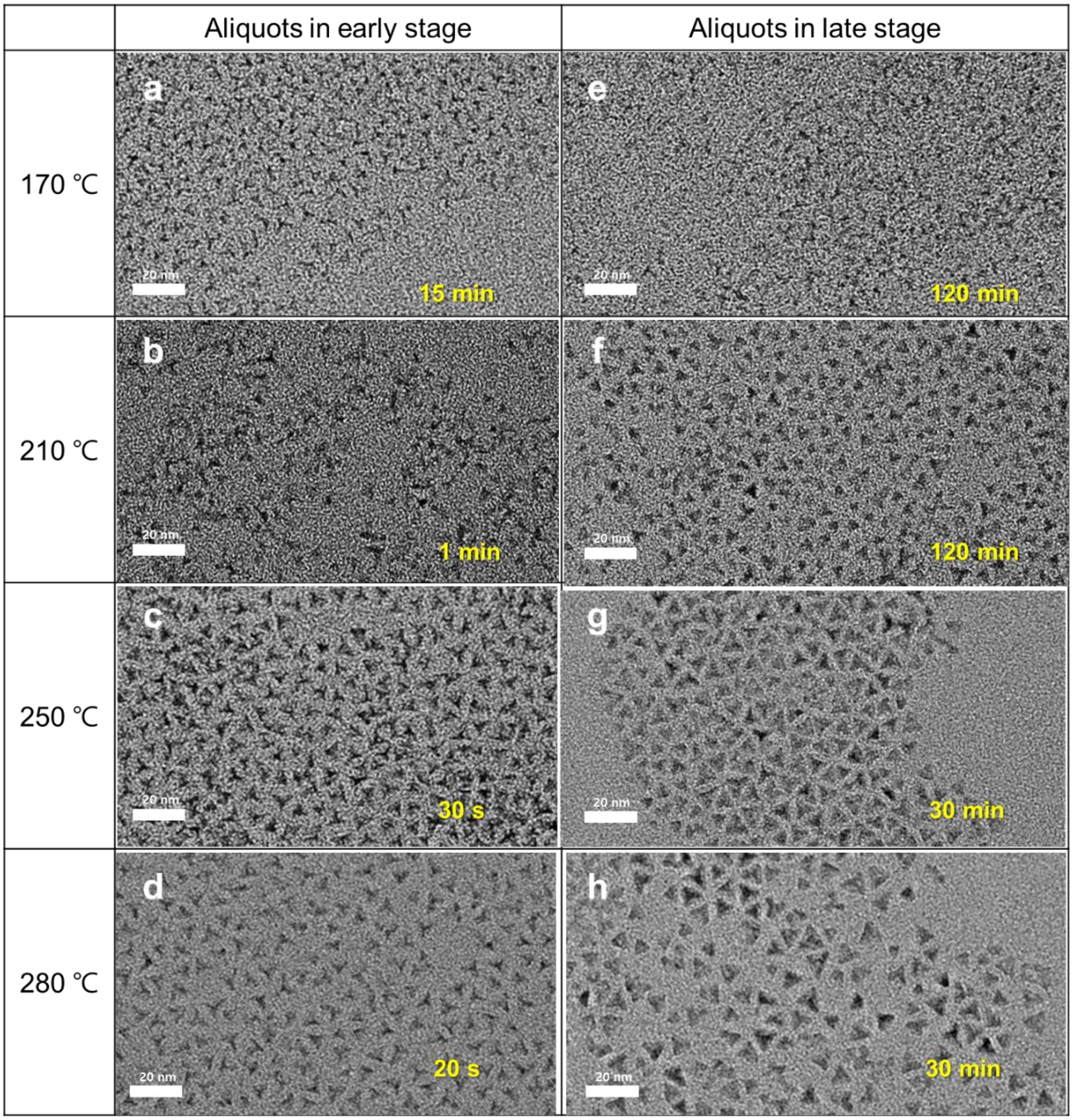

Supplementary Figure 2. TEM images of the early reaction stage of InP NCs synthesized at **a** 170, **b** 210, **c** 250, and **d** 280 °C, and the late reaction stage at **e** 170, **f** 210, **g** 250, and **h** 280 °C, respectively. In the aliquots from the early reactions, various tetrapod-like-shaped nanostructures are observed. In contrast, in the late reaction stage, tetrahedron InP NCs formed, except for the 170 °C reaction.

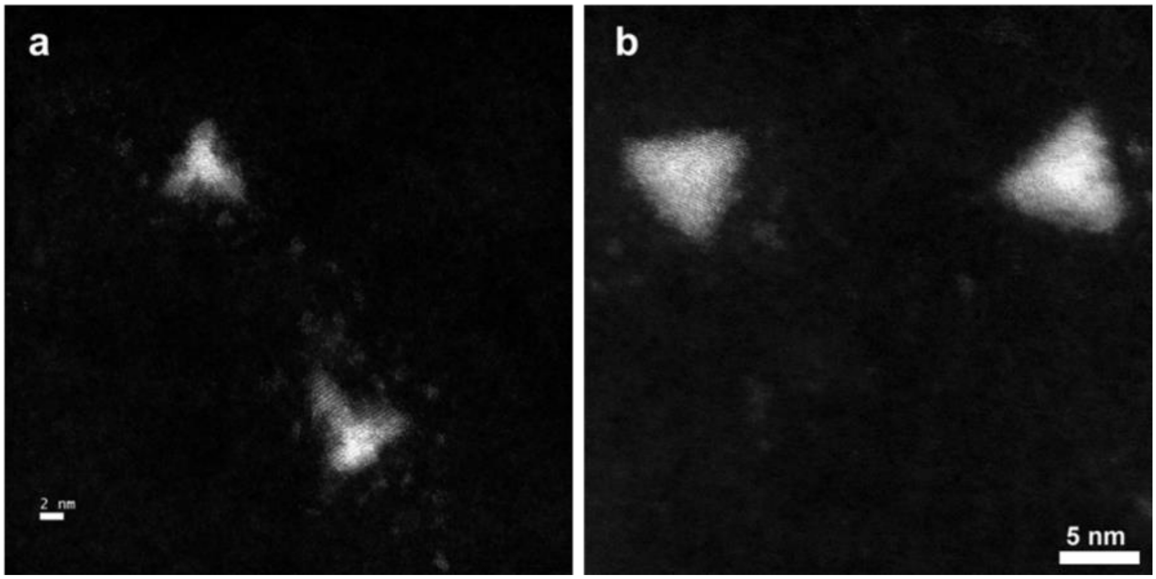

Supplementary Figure 3. Annular dark-field scanning TEM images of the **a** InP tetrapod and **b** tetrahedron NCs. The aliquots were taken from the reaction solution 5 s after tris(dimethylamino)phosphine injection at 300 °C. Tetrapod-like InP NCs clearly show the center arms as brighter spots in the middle.

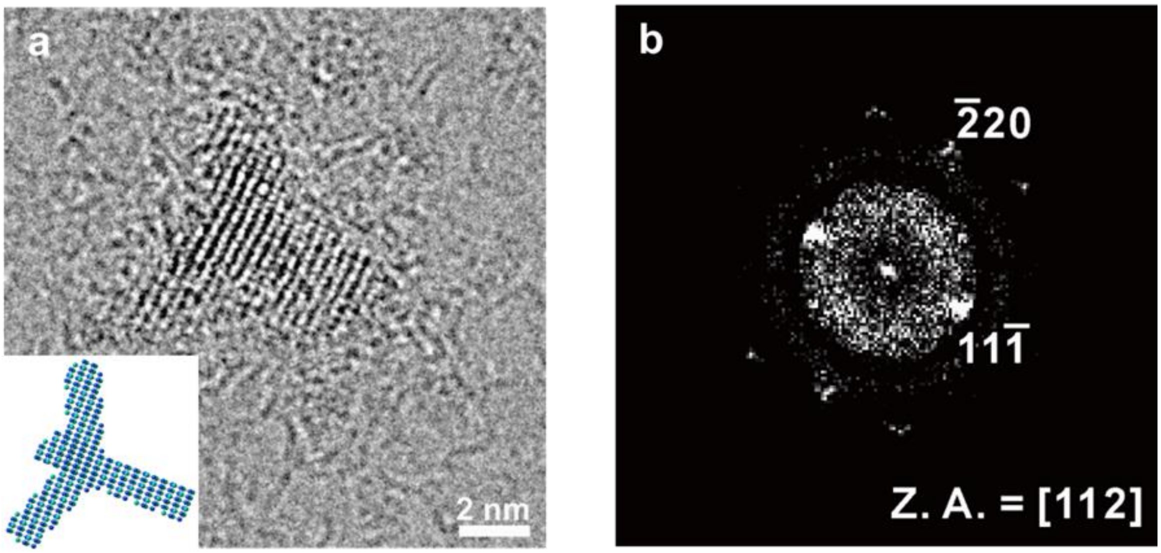

Supplementary Figure 4. a High-resolution TEM image of a tetrapod-like InP NC and **b** its corresponding fast Fourier transform pattern at the [112] zone axis. The measured sample was taken from the reaction solution 5 s after tris(dimethylamino)phosphine injection at 300 °C.

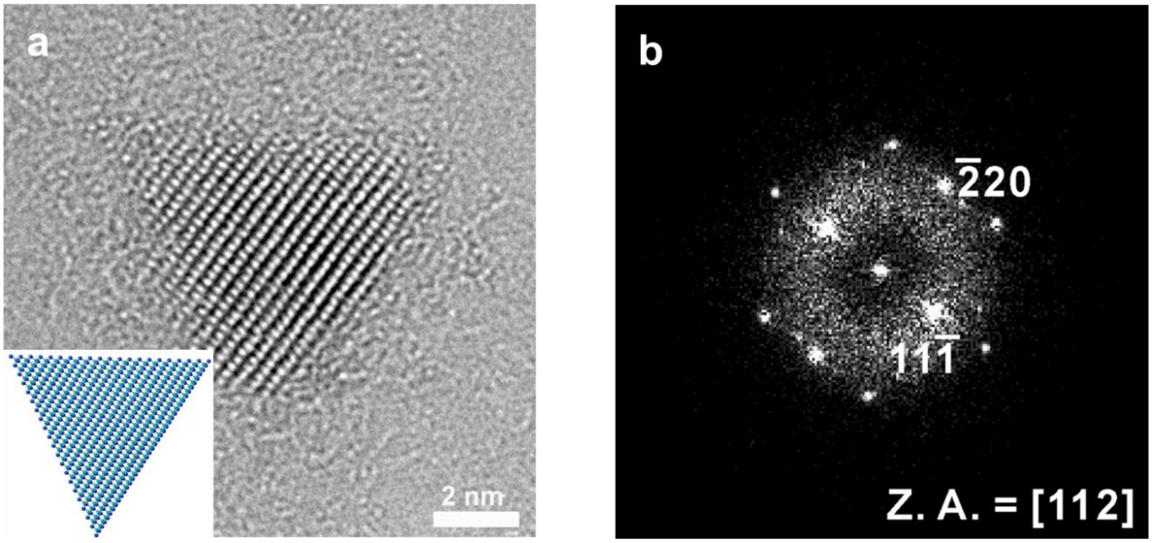

Supplementary Figure 5. a High-resolution TEM image of a tetrahedron-like InP NC and **b** its corresponding fast Fourier transform pattern at the [112] zone axis. The measured sample was taken from the reaction solution 5 s after tris(dimethylamino)phosphine injection at 300 °C.

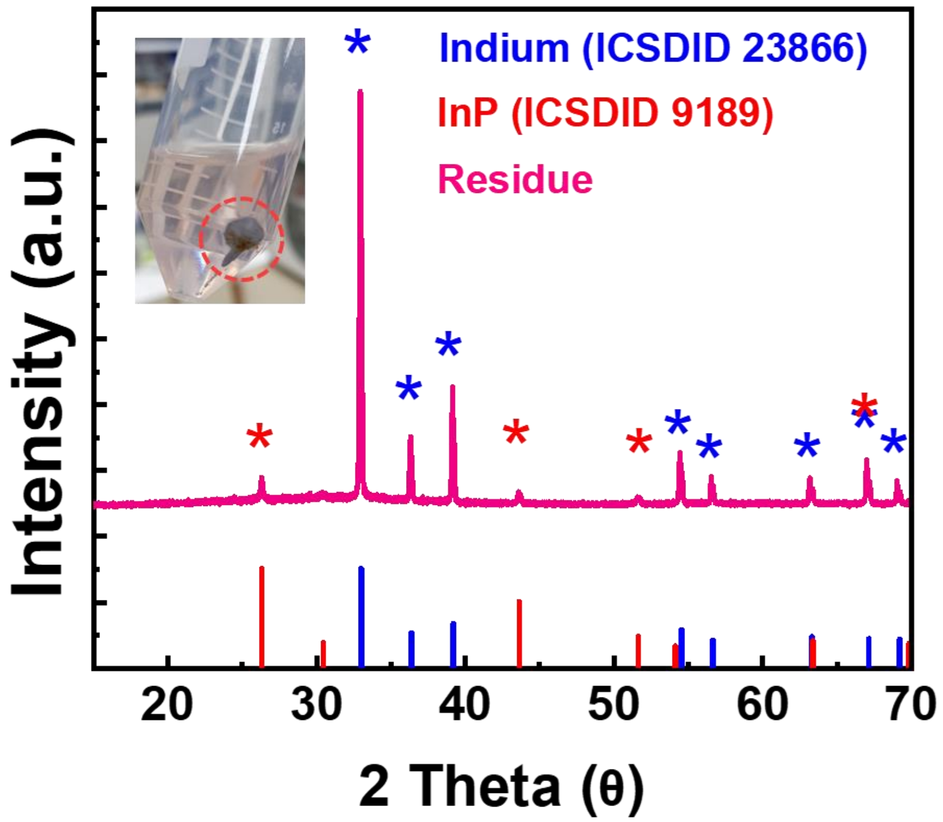

Supplementary Figure 6. X-ray diffraction (XRD) pattern of the residue of InP tetrapod synthesized using LiHMDS. The vertical lines represent bulk indium (blue, reference code - ICSD ID 23866) and bulk InP (red, reference code - ICSD ID 9189). The residue is mainly indium metal from the reduction of indium chloride and a small amount of InP.

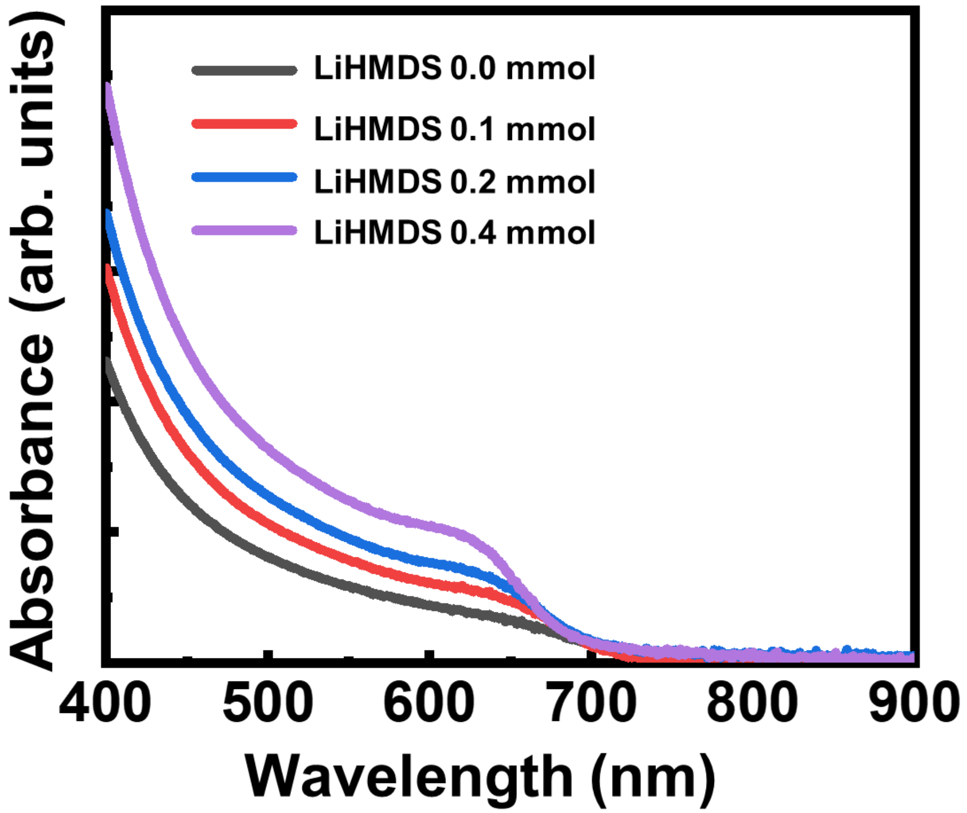

Supplementary Figure 7. Absorption spectra of the InP NCs synthesized using varied amount of LiHMDS (0.0–0.4 mmol of LiHMDS in 1.0 mmol of $InCl_3$). The synthesis was performed at 250 °C for 30 min. Excitonic peak positions were blue-shifted, and the optical density at 413 nm increased, as the amount of LiHMDS increase.

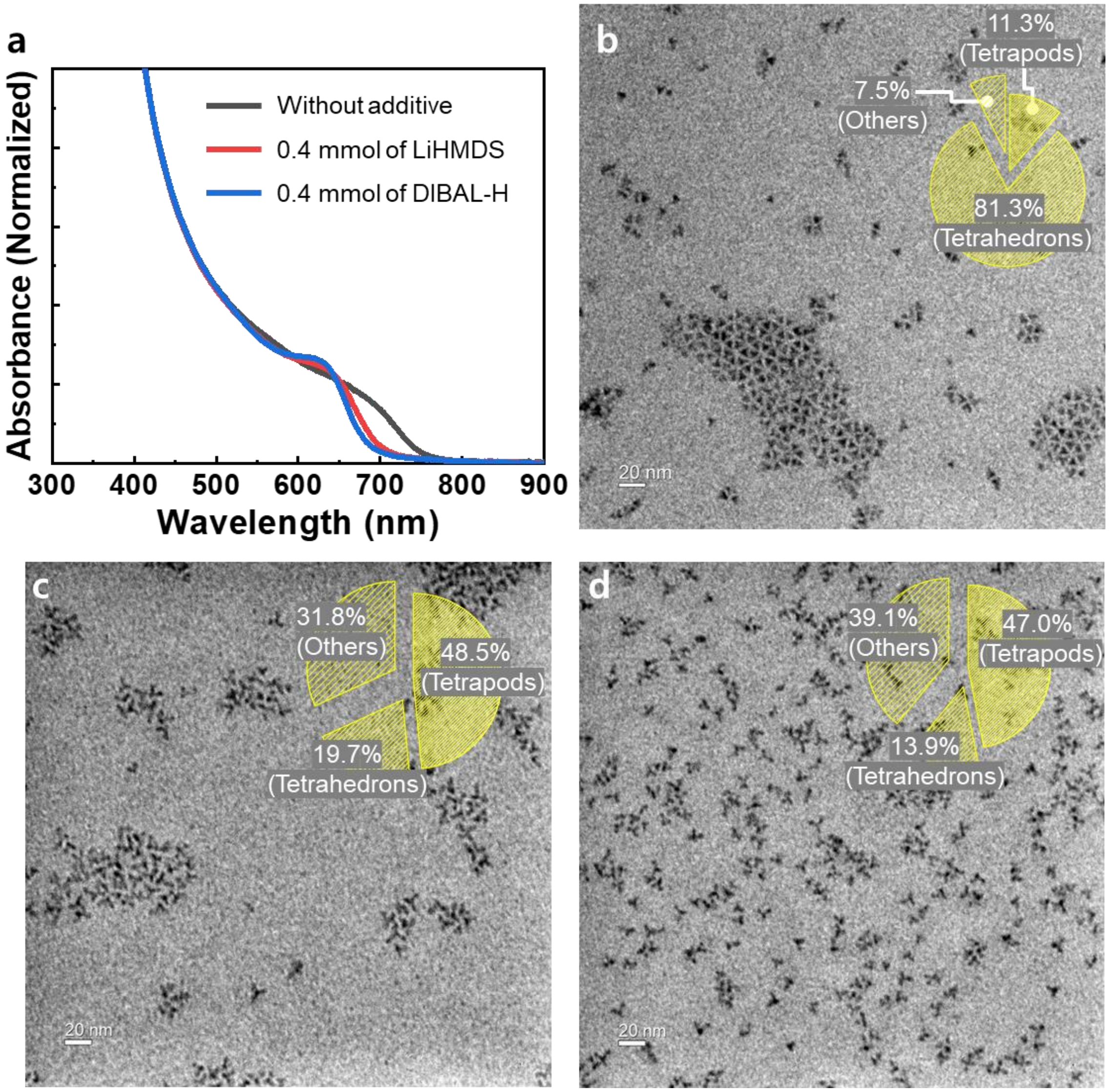

Supplementary Figure 8. a Absorption spectra of InP tetrapod NCs synthesized at 250 °C for 30 min in three different reaction conditions: 1) no additives (black line), 2) with 0.4 mmol LiHMDS (red line), and 3) with 0.4 mmol DIBAL-H (blue line). **b–d** TEM images of the InP tetrapod NCs corresponding to **b** no additives, **c** 0.4 mmol LiHMDS, and **d** 0.4 mmol DIBAL-H. Inset shows the pie chart of the shape yield. Shape yield of tetrahedron decrease from 81.3% to 19.7% with LiHMDS and 13.9 % with DIBAL-H, respectively.

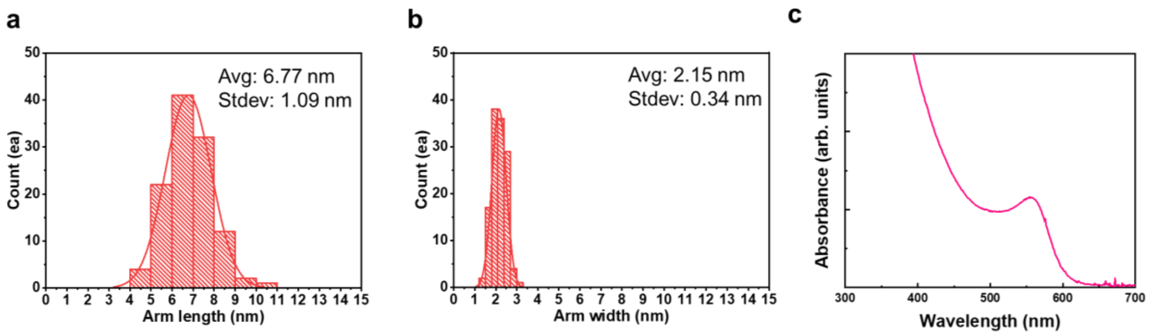

Supplementary Figure 9. Statistical analysis of the size uniformity of the isolated InP tetrapod NCs synthesized by the addition of LiHMDS at a ratio of 0.1 for LiHMDS to In at 170 °C for 60 min, corresponding to Figure 3a. Histograms of the **a** arm length, **b** width, and **c** absorption spectrum.

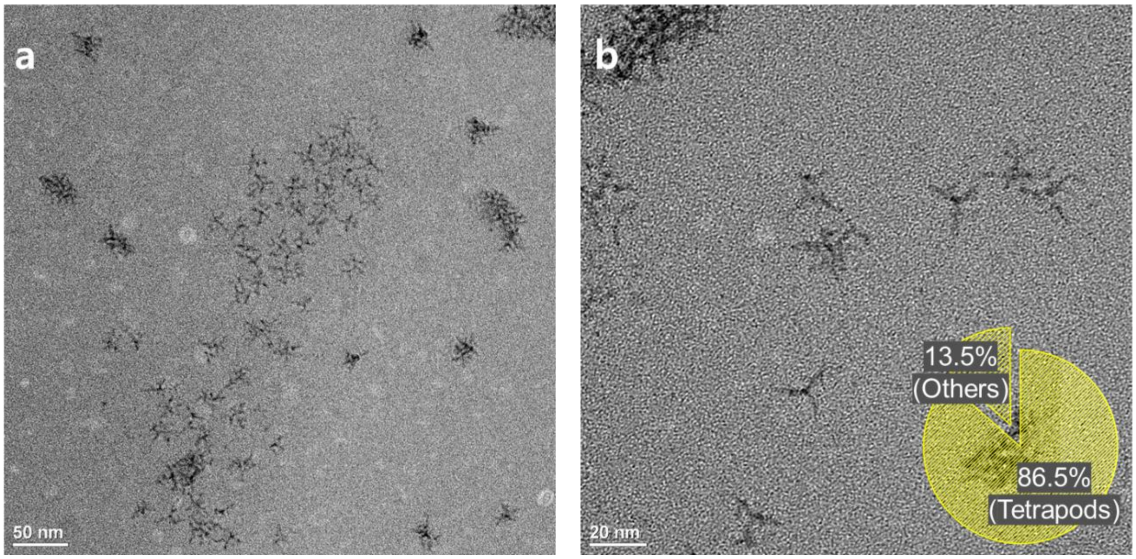

Supplementary Figure 10. a–b TEM images of the InAs tetrapod NCs synthesized by the addition of 0.1 mmol of LiHMDS when $(Me_2N)_3As$ was injected into an $InCl_3$ and OLA solution. The reaction proceeded at 220 °C for 60 min.

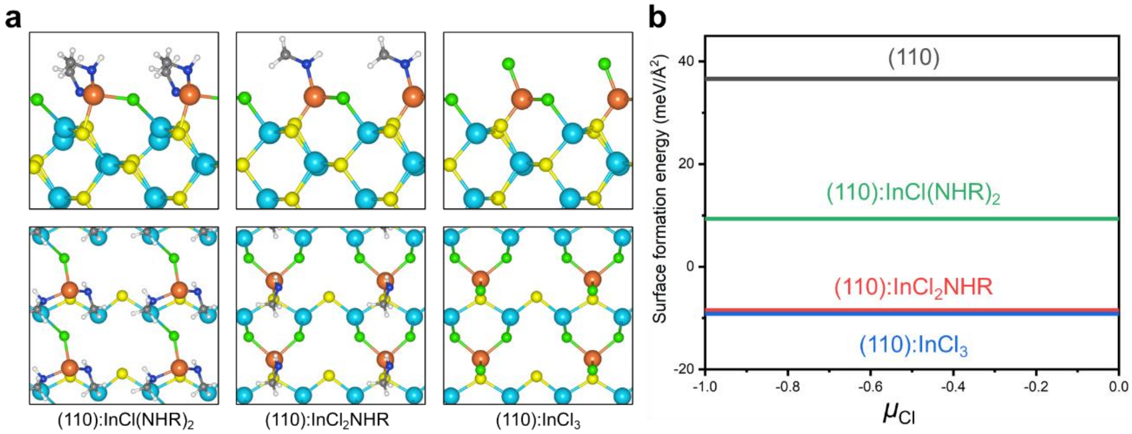

Supplementary Figure 11. **a** Side view (top) and top view (bottom) of the $InCl(NHR)_2$, $InCl_2NHR$ and $InCl_3$ ligand passivated (110) surfaces from left to right. The cyan, yellow, light green, blue, grey, white, and orange balls represent In, P, Cl, N, C, H and adsorbed In atoms, respectively. **b** Surface formation energy of the ligand passivated (110) facet. In the case of $InCl_3$ and $InCl_2NHR$ passivated (110) surfaces, two Cl atoms passivate two surfaces In atoms, and the remaining InCl and InNHR moieties passivate two surface P atoms while it binds to a single P atom. On the hand, $InCl(NHR)_2$ ligand passivates one surface In atom and one surface P atom. Due to unpassivated surface atoms, $InCl(NHR)_2$ ligand passivated (110) surface is less stabilized than two Cl atoms contained molecule passivated (110) surfaces.

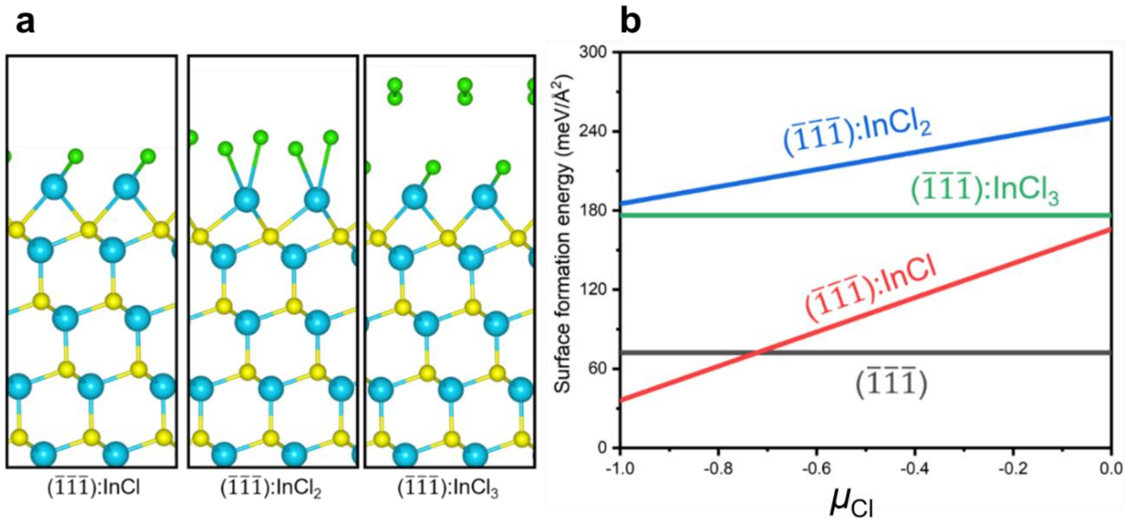

Supplementary Figure 12. **a** Side view of the relaxed $(\bar{1}\bar{1}\bar{1})$ surfaces passivated by InCl, $InCl_2$, and $InCl_3$ from left to right. The cyan, yellow, and light green represent indium, phosphorus, and chlorine atoms, respectively. **b** Surface formation energy of the $(\bar{1}\bar{1}\bar{1})$ facets passivated with $InCl_x$. The InCl passivation is favored over $InCl_2$ and $InCl_3$ due to their steric hindrance.

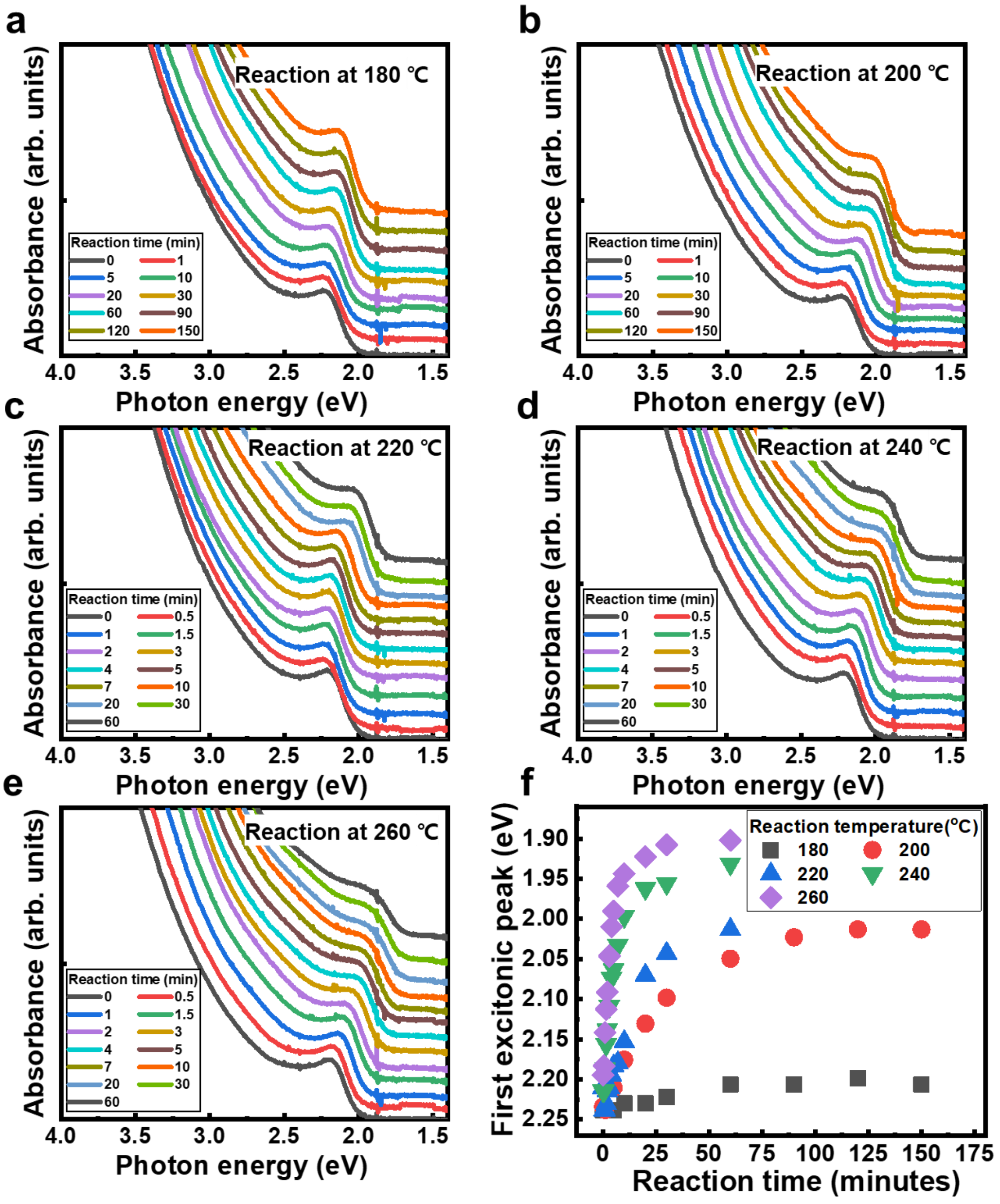

Supplementary Figure 13. To obtain the activation energy of [110] growth, absorption spectra were measured as the excitonic peak shift represents [110] growth. **a**–**e** Absorption spectra of the InP tetrapod NCs with additional precursor injection at various temperatures (180–260 °C). The aliquots were taken at various reaction times. **f** Temperature-dependent evolution of the first exciton peak as a function of the growth time, obtained from **a**–**e**.

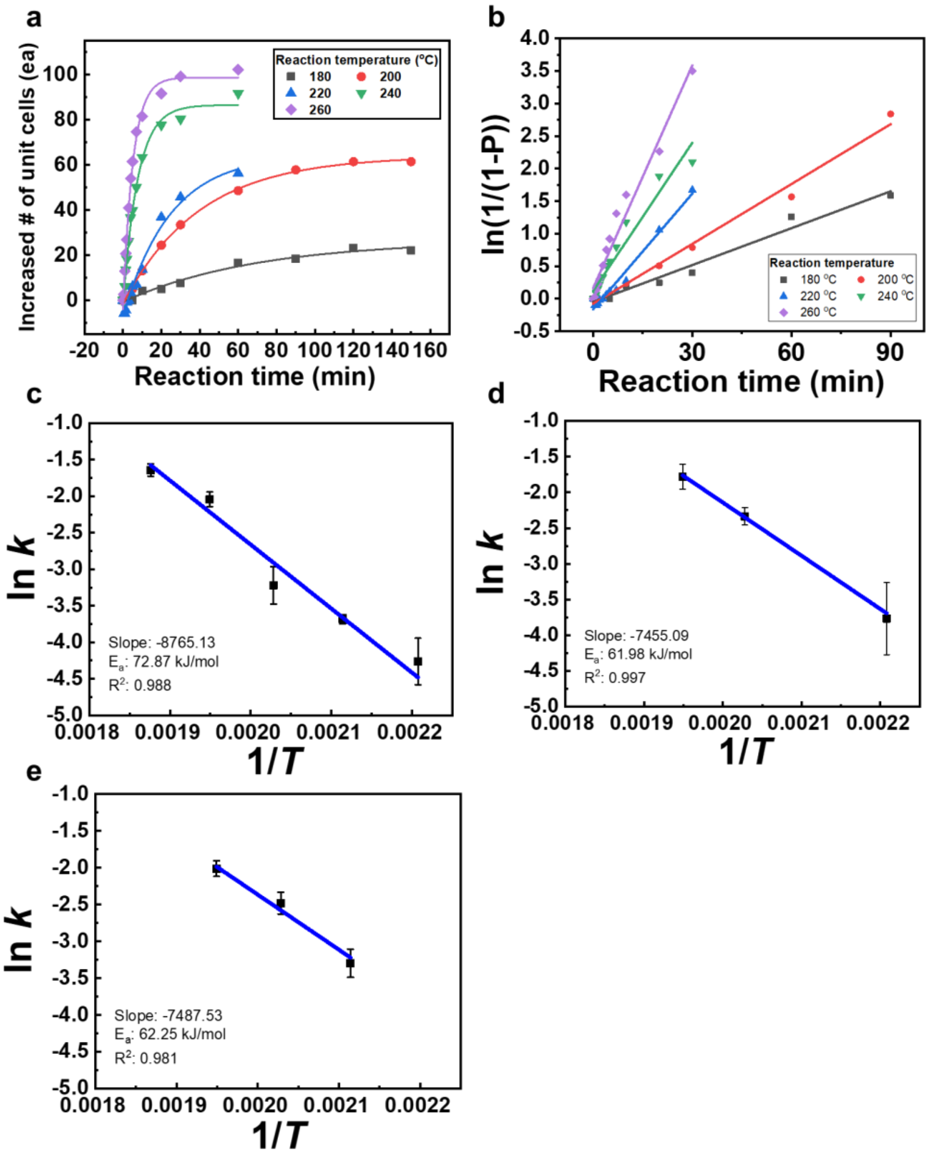

Supplementary Figure 14. a Plot of the increased number of InP unit cells as a function of the reaction time, which were converted from Supplementary Fig. 13f. The rate constant, k, was extracted from this plot. **b** Linearly fitted plot of ln(1/(1-P)) versus reaction time (min) indicates that the order of reaction is one. **c** Plot of ln k versus 1/T, extracted from **a**. The slope of this plot is the same as E_a/R, where E_a is the activation energy for the [110] growth and R is the gas constant. The activation energy, E_a, was determined as 72.87 kJ/mol. **d–e** Two independent experimental results to confirm the reproducibility. The average activation energy value was 65.7 ± 6.2 kJ/mol. Error bars represent the standard deviation of ln *k* values which extracted from absorbance spectra.

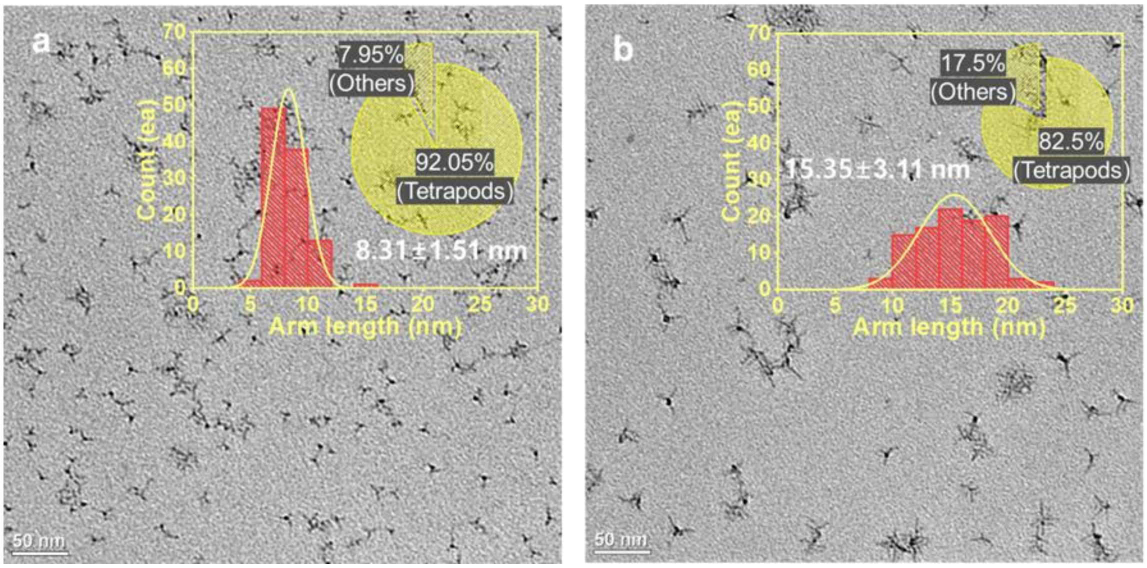

Supplementary Figure 15. TEM images of the **a** isolated InP tetrapod intermediates used as a growth platform and **b** $(\bar{1}\bar{1}\bar{1})$-grown InP tetrapods synthesized by supplying additional precursor ($InCl_3$ and $P(NMe_2)_3$) into **a**. Insets in **a**–**b** are the histogram of the arm length and pie chart showing the shape yield in each TEM image. When the tetrapods become longer, we often observe a smaller tetrapod shape yield possibly from increased aggregation.

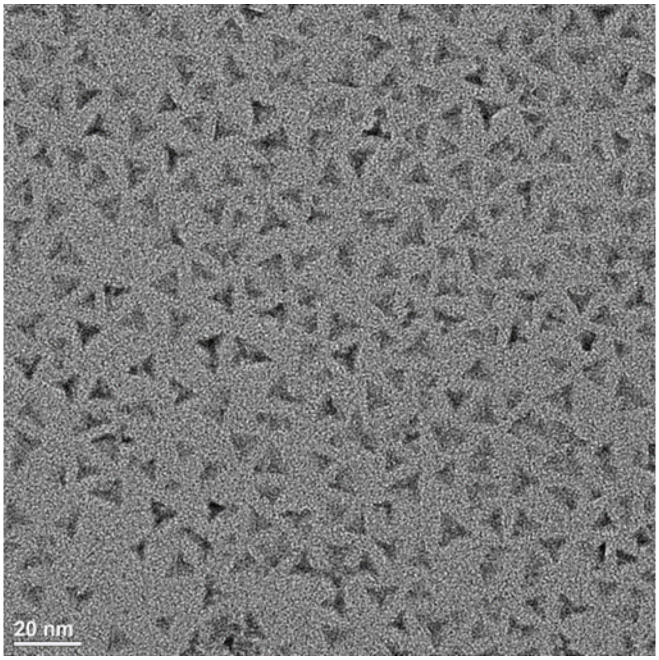

Supplementary Figure 16. TEM image of the InP NCs grown by supplying additional precursors ($InCl_3$ and $P(NMe_2)_3$) into the tetrapod intermediates at 250 °C.

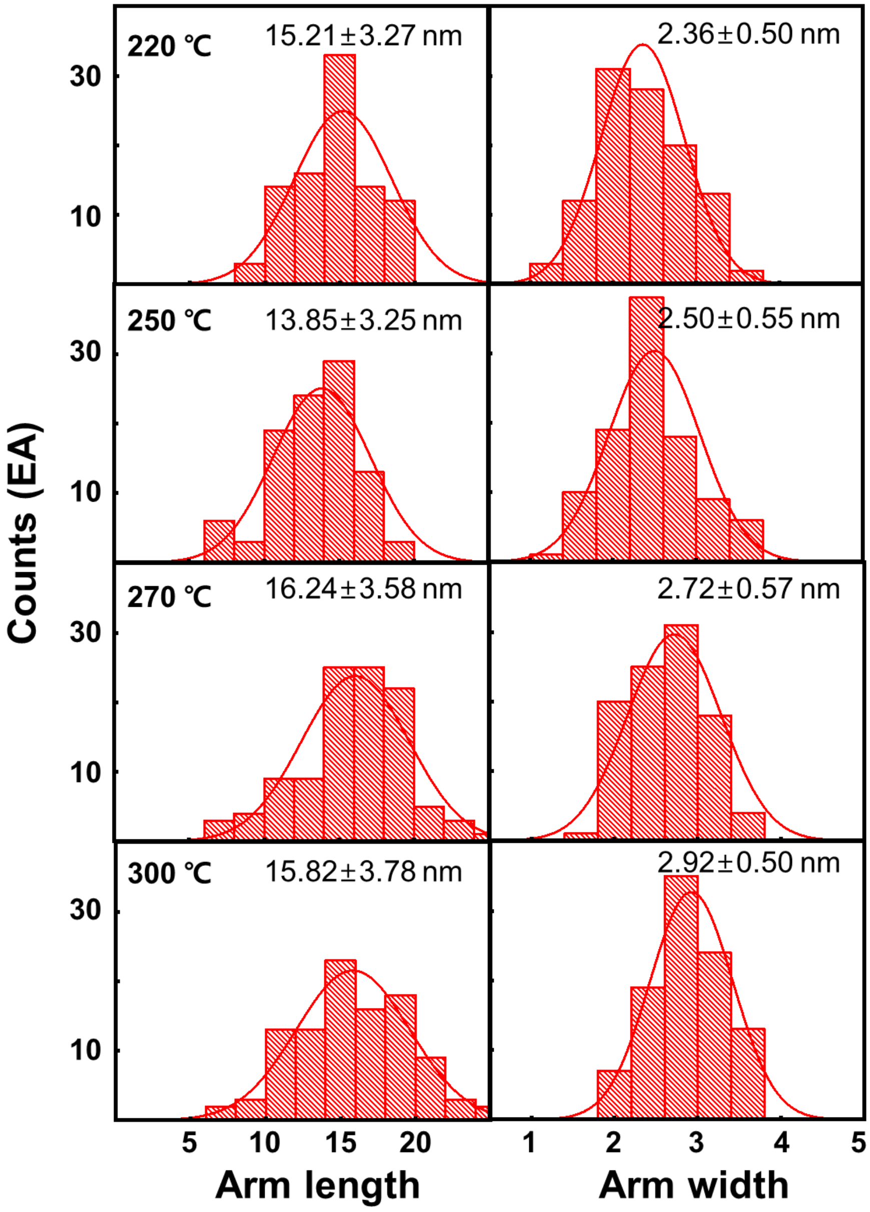

Supplementary Figure 17. Arm-length and arm-width histograms of the [$\bar{1}\bar{1}\bar{1}$]/[110] grown InP tetrapods matched with the short dashed line in Figure 5a. The shift in the excitonic peaks in Figure 5a is increased with the growth of arm-width depending on the heating temperature (details in Supplementary Fig. 18a).

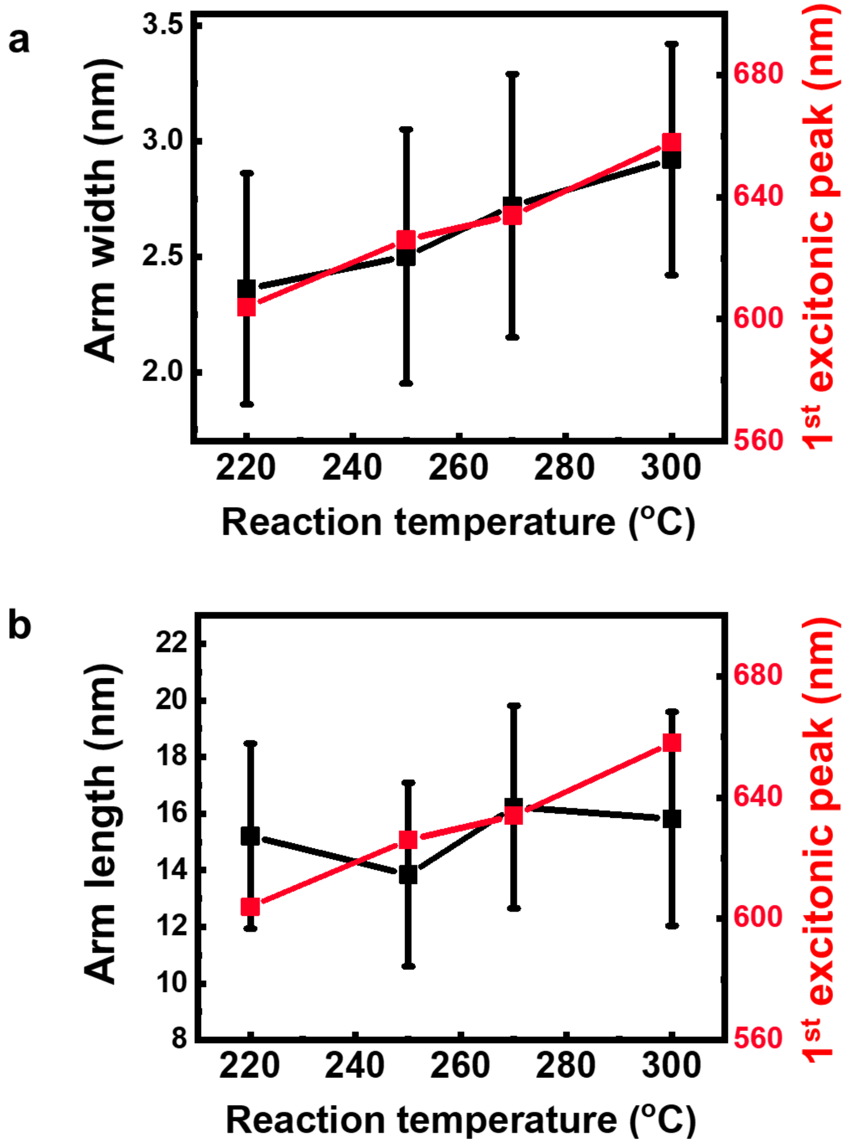

Supplementary Figure 18. Correlation of the **a** arm-width and **b** -length with the first excitonic peak of the InP tetrapod NCs at various temperatures. Error bars are the standard deviation extracted from measured particle sizes. The data were obtained from Supplementary Fig.17 and Figure 5a.

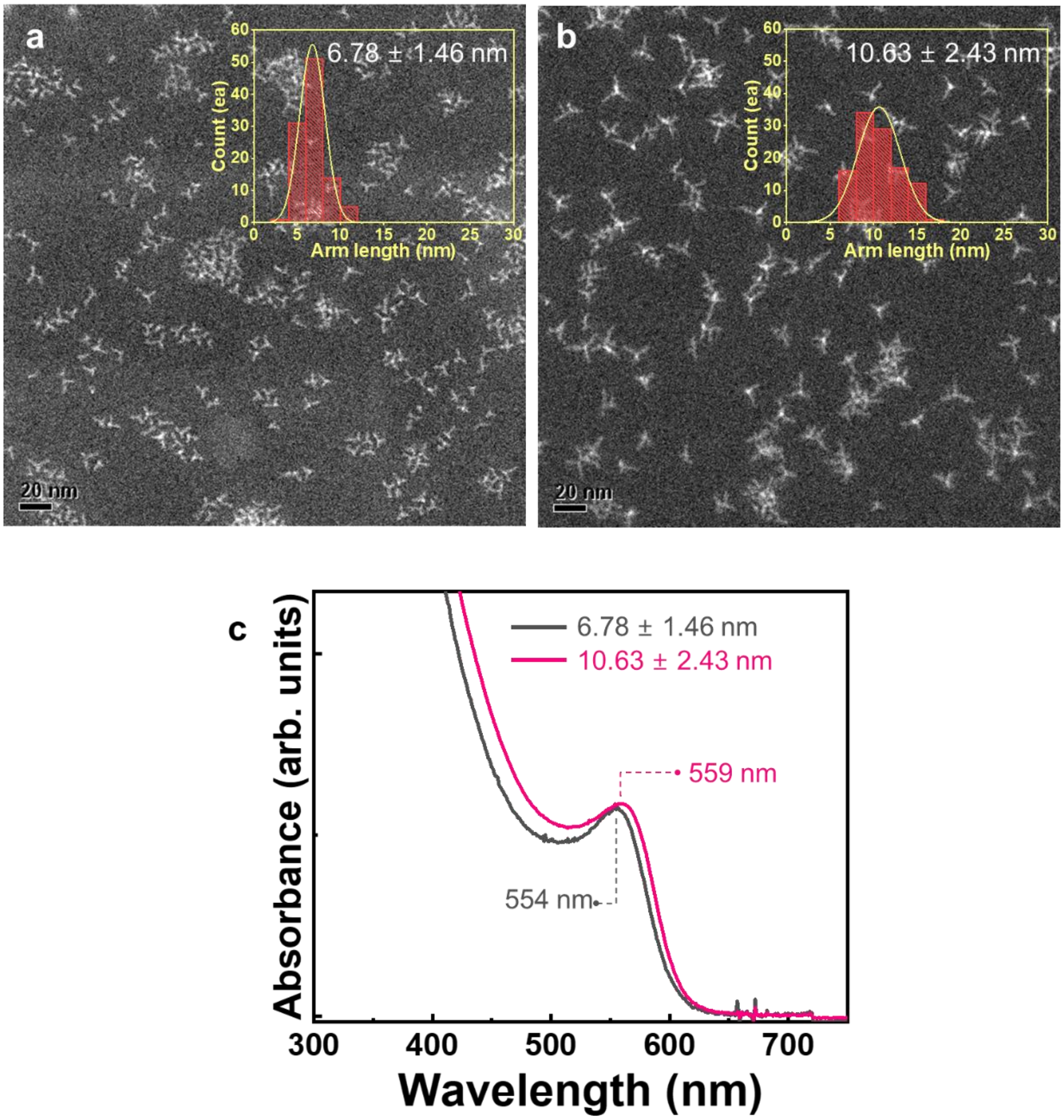

Supplementary Figure 19. TEM images of the InP tetrapod NCs with **a** short and **b** long arms. **c** Absorption spectra of the InP tetrapod NCs with short and long arms. Although the arm length of the tetrapod was changed by ~4 nm, the excitonic peak position changed only slightly (red shift of ~5 nm), which was different from the arm-width dependent excitonic peak shift, as shown in Supplementary Fig. 18

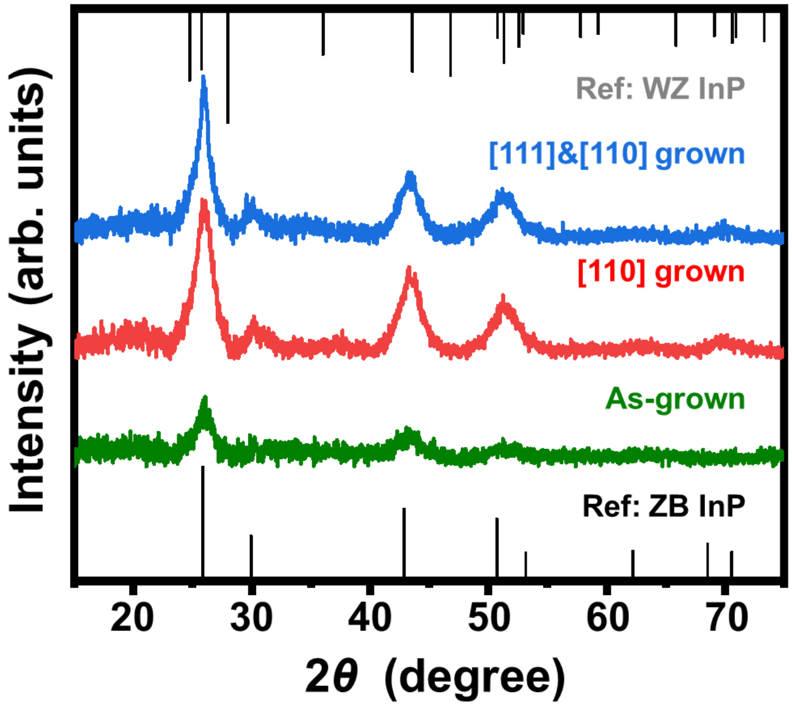

Supplementary Figure 20. Powder X-ray diffraction patterns of various tetrapod NCs with additional growth along the [110] and [$\bar{1}\bar{1}\bar{1}$] directions. The standard X-ray diffraction pattern of the zinc blende (ZB) InP crystal (Reference code: 98-016-5466) and the wurtzite (WZ) InP crystal (Reference code: 98-018-0911) are displayed for reference.

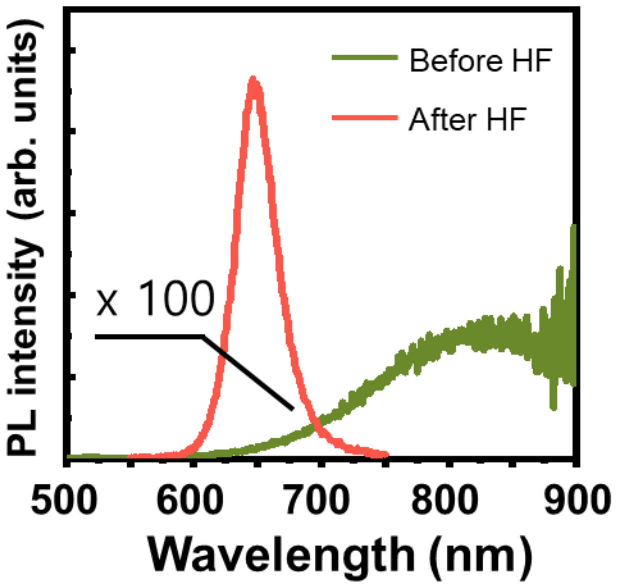

Supplementary Figure 21. Photoluminescence spectra of the InP tetrapods **a** before and **b** after the HF treatment. After the HF treatment, the InP tetrapods show enhanced band-edge emission with fully suppressed trap emission.

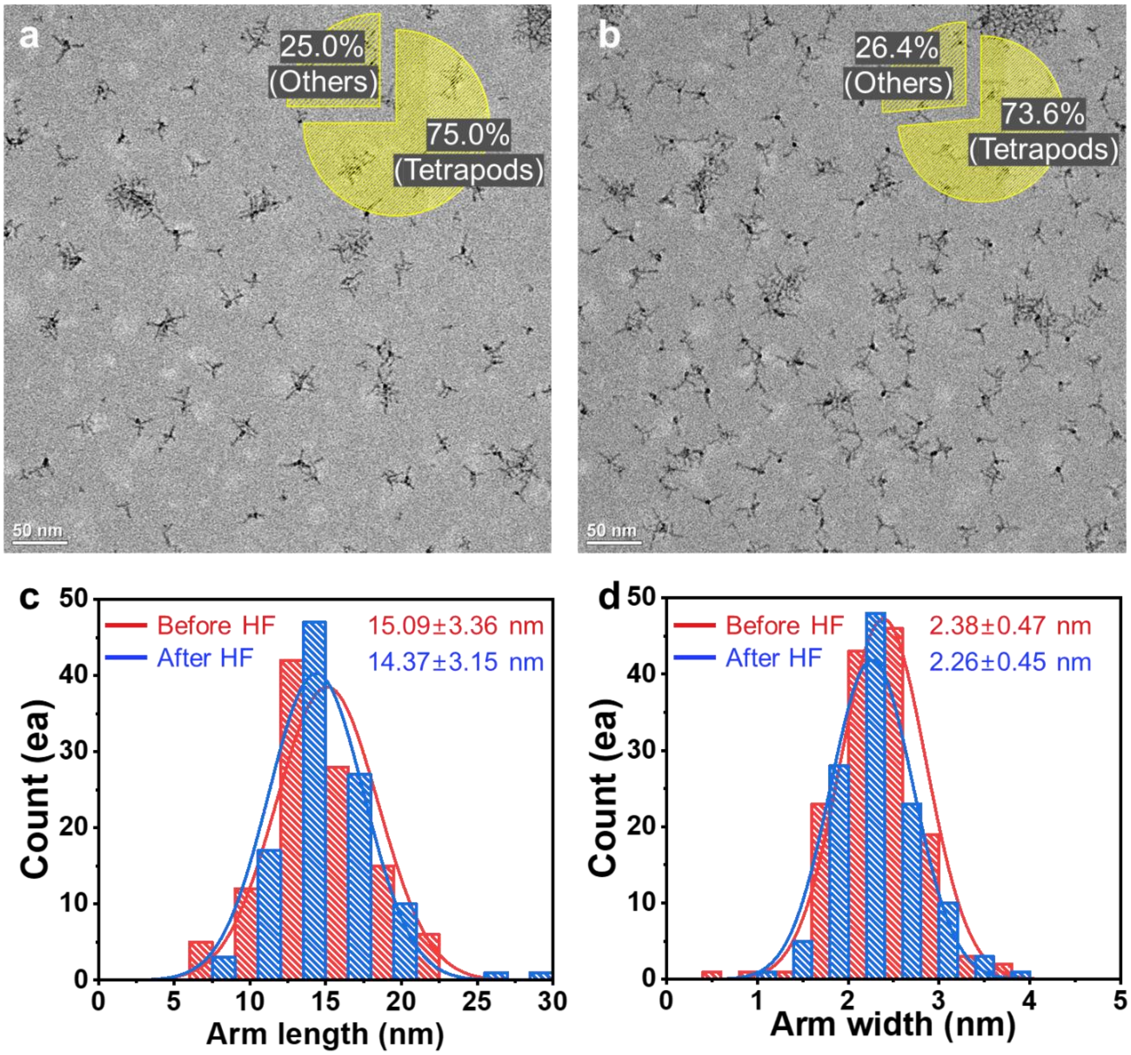

Supplementary Figure 22. TEM images of the [$\bar{1}\bar{1}\bar{1}$] and [110] grown InP tetrapods **a** before HF treatment and **b** after HF treatment for 190 min. **c–d** Histogram of the length and width of the arm for comparison **c** before and **d** after HF treatment. Others in the inset of **a-b** included agglomerated tetrapods which are not corresponding to the view angle dependent tetrapod shape.

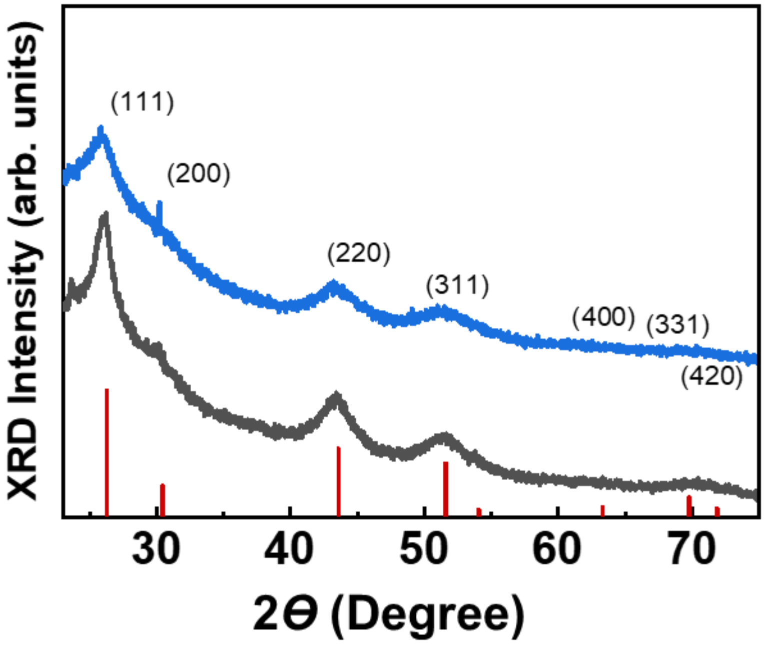

Supplementary Figure 23. X-ray diffraction (XRD) patterns of the [110] and [$\bar{1}\bar{1}\bar{1}$] grown InP tetrapod NCs before (black) and after (blue) HF treatment. The vertical line on the bottom represents the bulk InP zinc blende structure (Reference - ICSD ID 9189). Crystal structure of the InP tetrapods remained as a zinc-blende structure after HF treatment.

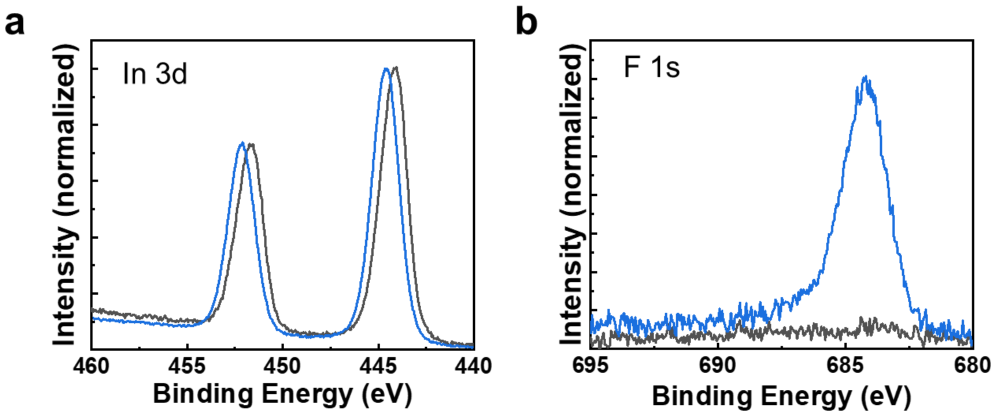

Supplementary Figure 24. XPS analysis of the InP tetrapods before (black line) and after HF treatment (blue line). **a** The In 3d spectrum shows the high binding energy shift after HF treatment, which implies the formation of In-F or In-O. **b** The intensity of the F 1s spectrum increased after the HF treatment, which implies F passivation on the InP surface.

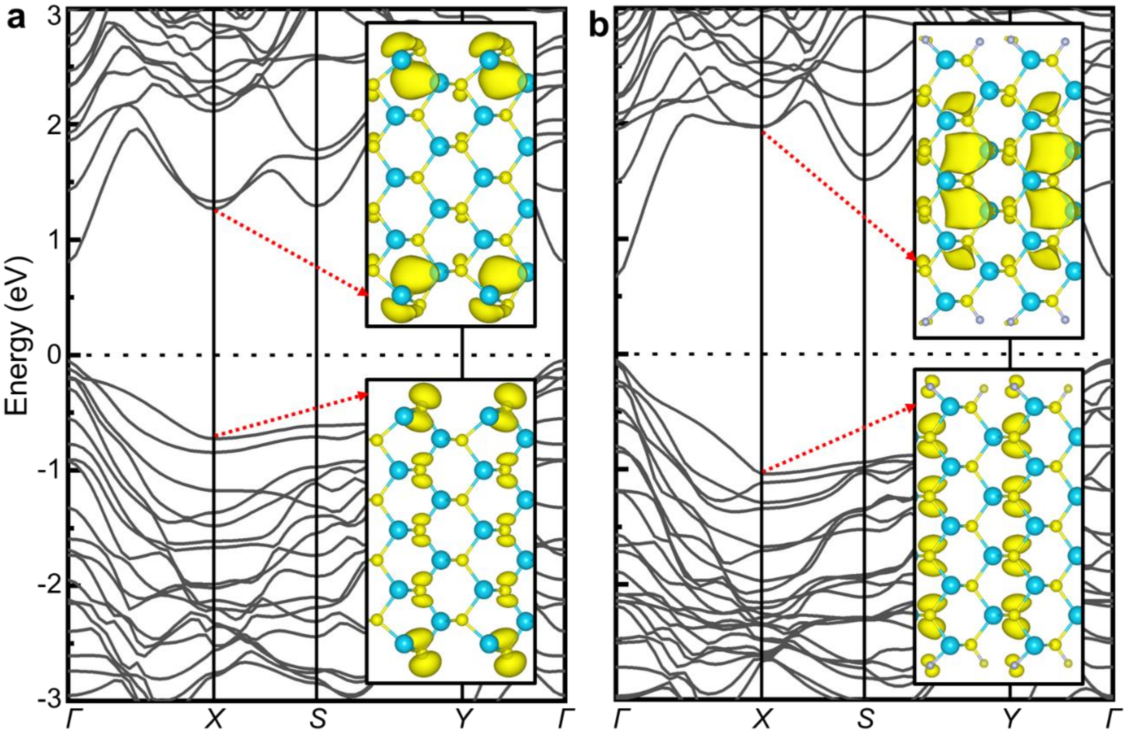

Supplementary Figure 25. Band structure of **a** self-passivated and **b** F-passivated (110) surfaces. The insets are charge-density plots of the highest occupied state and lowest unoccupied state at the X-point. The surface states of the reconstructed (110) facet at the X-point are removed into the bulk states by the passivation of fluorine. The cyanine, yellow, lavender balls represent indium, phosphorus, and fluorine atoms, respectively.

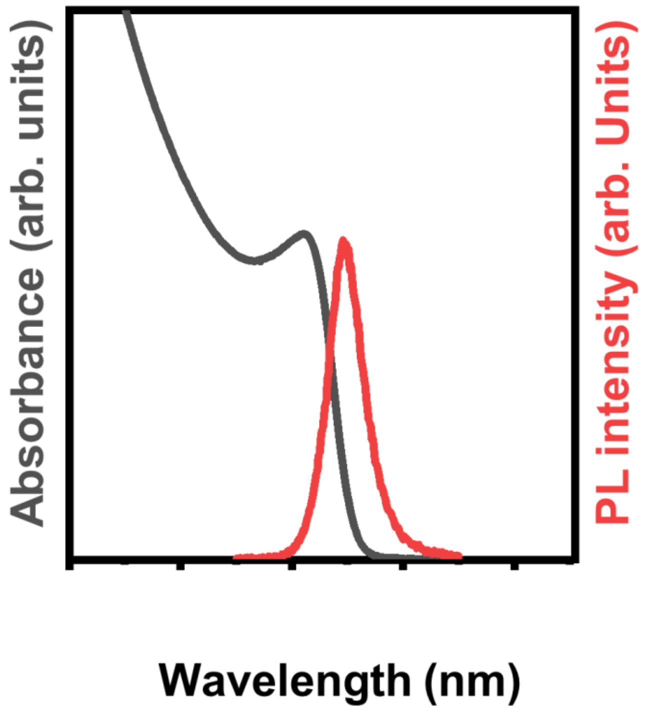

Supplementary Figure 26. Absorption and photoluminescence spectra of the HF-treated InP tetrapod NCs, which have a 35.9 nm FWHM value at 646 nm of PL maximum.

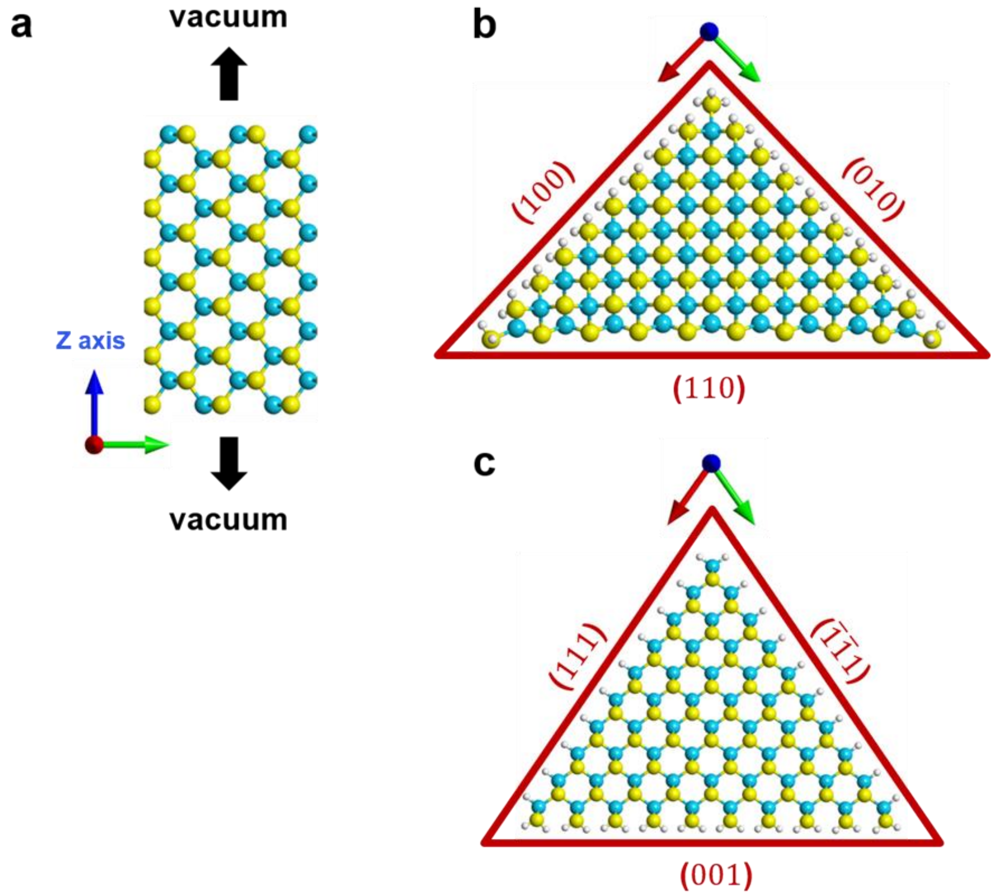

Supplementary Figure 27. a Side view of the bare (110) surface slab model with the vacuum layer along the z-axis. **b–c** Triangular cross-sections of the wedge model consisting of two equivalent surfaces. The blue, green, and white balls represent indium, phosphorus, and hydrogen atoms, respectively.

Supplementary Table 1. DFT absolute surface energies (γ, meV/Å^2) of the bare (100), (110), (111), and $(\bar{1}\bar{1}\bar{1})$ surfaces of InP obtained from the wedge and slab models.

Surface	Surface energy (meV/Å^2)
(100)	85.1
(110)	36.6
(111)	72.3
$(\bar{1}\bar{1}\bar{1})$	73.4

Supplementary Table 2. DFT surface formation energy (γ, meV/Å^2) of the (110), (111), and $(\bar{1}\bar{1}\bar{1})$ surfaces passivated with chlorine (Cl), methylamine (MA), oleylamine (OLA), $InCl_3$, $InCl_2NHCH_3$, and InCl.

Surface	Ligand	Surface formation energy (meV/Å^2)
(110)	$InCl_3$ (2 × 1)	–9.2
	$InCl_2NHCH_3$ (2 × 1)	-8.5
(111)	3Cl, MA (2 × 2)	–75.7
	3Cl, OLA (2 × 2)	–76.8
	Cl (1 × 1)	–58.7
$(\bar{1}\bar{1}\bar{1})$	InCl (1 × 1)	165.9

Supplementary Table 3 Elemental ratios of InP tetrapods and tetrahedrons obtained from X-ray photoemission spectroscopy analyses.

	Tetrapod (short arm)	Tetrapod (long arm)	Tetrahedron
In/P	1.3 ± 0.1	1.2 ± 0.1	1.5 ± 0.1
Cl/In	0.5 ± 0.1	0.4 ± 0.1	0.3 ± 0.1
N/In	0.5 ± 0.1	0.6 ± 0.2	0.4 ± 0.1

Supplementary Note 1| Measurement of length and width of tetrapods depending on the view angle.

We suggested the view-angle dependent shape in Figure 3b. The length of tetrapod's arms can also differently appear depending on the view-angle. Therefore, we define length of tetrapod's arm here. (Arm width, w, of tetrapod was not changed by viewing angle thus, we can measure width of all tetrapod except particles agglomerated and overlapped regime in TEM images.)

The length, l, of tetrapod was defined as the distance of tetrapod's center to a vertex. In [112] and [110] zone axes, the tetrapod's arms can stand parallel to the floor, thus we can measure length directly. On the other hand, in [111], [100] zone axes, we have to consider the arms inclined and we must convert measured length to actual length. In addition, other various view-angle such as [230] can vary the measured l and the actual l and we cannot distinguish small changes of angle. Therefore, we only measure length of tetrapod in [112], [110], [111], and [100] zone axes. In the measurement of the shape yield, we consider all angle dependent tetrapod shape. Considering this, we suggested converting the value of the length in tetrapod below:

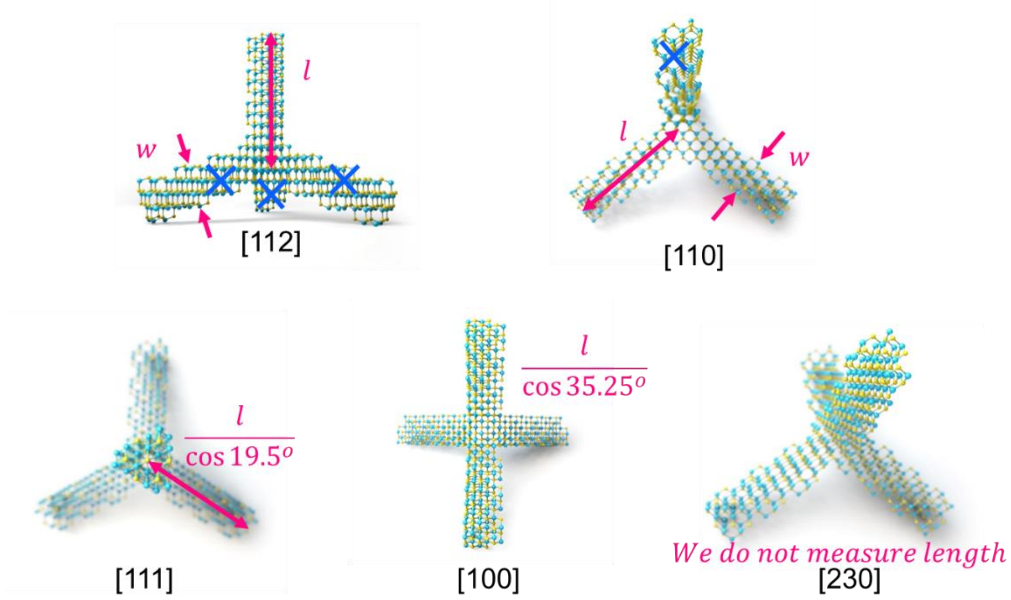

Supplementary Note 2| Determination of the activation energy barrier for [110] growth

Assumption for activation energy measurement on the [110] growth

a. InP tetrapod grows into tetrahedron via [110] direction growth. (Although complex chemistry may exist in tetrapod to tetrahedron transition, the [110] growth of tetrapod is considered as main reaction step for tetrahedron transition.)

b. 1st excitonic peak in the absorbance spectra is predominantly determined by the [110] grown width, rather than the $[\bar{1}\bar{1}\bar{1}]$ grown length of the arms.

c. Optical bandgap can be converted by arm width by 2D-confinemed InP nanocrystal model.[1]

Extraction of the rate constant "k" as a function of the increased number of InP unit cells with the reaction time

Based on the absorbance spectra for various reaction temperatures, we extracted the first excitonic peaks (units of electron volts). As the first excitonic peaks of the absorption spectra strongly depend on the arm width of the tetrapod, which reflects [110] growth, we used the first excitonic peak of the absorption spectra to determine the [110] growth rate. The first excitonic peaks were converted into the diameter of the tetrapod arm using the theoretical 2-D confinement equation for the InP nanowire[1]:

$$E_g = 1.35 + \frac{3.8}{d^2} \tag{1}$$

where E_g is the bandgap energy of the tetrapod and d is the diameter of the tetrapod arm. The arm of the tetrapod was assumed to have an equilateral triangular prism shape, which was exposed by three (110) facets. The volume of the InP tetrapod was obtained by calculating the volume of an equilateral triangular prism:

$$V = \frac{\sqrt{3}}{4} * d^2 * h * 4 \tag{2}$$

where h is the length of the tetrapod arm.

The increased volume of the tetrapod was obtained as follows:

$$V_{\text{increased}} = V_t - V_0 \tag{3}$$

where V_t is the volume at reaction time t and V_0 is the volume at a reaction time of 0 min.

The number of increased InP unit cells was calculated as follows:

$$N_{\text{unit cells}} = \frac{V_{\text{increased}}}{V_{\text{unit cell}}} \quad (4)$$

Supplementary Fig. 14a shows the number of increased unit cells in the InP tetrapod as a function of the reaction time. To confirming the reaction order, we checked increased product versus reaction time plot. As a result, we confirmed the linear plot which means our reaction well-matched with 1st-order reaction. (Supplementary Fig. 14b)[2] The rate constant k was extracted from the increased number of InP unit cells in the reaction time plot (Supplementary Fig. 14a) using the following first-order reaction equation:

$$y = A * (1 - e^{-kx}) + C \quad (5)$$

Where y is the number of produced InP units, A is the pre-exponential factor, k is the rate constant, x is the reaction time, and C is the initial number of InP unit cells.

Extraction of the activation energy barrier (E_a)

The extracted k value was converted into an ln k versus 1/T plot to obtain the activation energy barrier. Based on the Arrhenius equation, i.e., $k = Ae^{(-Ea/RT)}$, where A is a constant, R is the gas constant in J/mol·K, and T is the reaction temperature, the slope in the ln k versus 1/T plot is E_a/R, where E_a is the activation energy and R is the gas constant. Thus, we obtained an activation energy of 72.87 kJ/mol. To confirm the reproducibility, we repeated the experiments and calculations three times, and the average activation energy was obtained as 65.7 ± 6.2 kJ/mol. (Supplementary Fig. 14)

Supplementary References

[1] Yu, H., Li, J., Loomis, R. A., Wang, L.-W. & Buhro, W. E. Two- versus three-dimensional quantum confinement in indium phosphide wires and dots. *Nat. Mater.* **2**, 517–520 (2003).

[2] Zhou, Y. et al. Quantitative nucleation and growth kinetics of gold nanoparticles via model-assisted dynamic spectroscopic approach. *J. Colloid Interface Sci.* **407**, 8–16 (2013).
